# Can ChatGPT provide high-quality patient information on male lower urinary tract symptoms suggestive of benign prostate enlargement?

**DOI:** 10.1038/s41391-024-00847-7

**Published:** 2024-06-13

**Authors:** Angie K. Puerto Nino, Valentina Garcia Perez, Silvia Secco, Cosimo De Nunzio, Riccardo Lombardo, Kari A. O. Tikkinen, Dean S. Elterman

**Affiliations:** 1 Faculty of Medicine, University of Helsinki, Helsinki, Finland; 2 Division of Urology, Department of Surgery, University of Toronto, Toronto, ON Canada; 3 Faculty of Medicine, University of the Andes, Bogota, Colombia; 4 Department of Urology, Niguarda Hospital, Milan, Italy; 5 Urology Unit, Ospedale Sant’Andrea, La Sapienza University of Rome, Rome, Italy; 6 Department of Urology, University of Helsinki and Helsinki University Hospital, Helsinki, Finland; 7 Department of Surgery, South Karelian Central Hospital, Lappeenranta, Finland; 8 Department of Health Research Methods, Evidence and Impact, McMaster University, Hamilton, ON Canada

**Keywords:** Translational research, Outcomes research

## Abstract

**Background:**

ChatGPT has recently emerged as a novel resource for patients’ disease-specific inquiries. There is, however, limited evidence assessing the quality of the information. We evaluated the accuracy and quality of the ChatGPT’s responses on male lower urinary tract symptoms (LUTS) suggestive of benign prostate enlargement (BPE) when compared to two reference resources.

**Methods:**

Using patient information websites from the European Association of Urology and the American Urological Association as reference material, we formulated 88 BPE-centric questions for ChatGPT 4.0+. Independently and in duplicate, we compared the ChatGPT’s responses and the reference material, calculating accuracy through F1 score, precision, and recall metrics. We used a 5-point Likert scale for quality rating. We evaluated examiner agreement using the interclass correlation coefficient and assessed the difference in the quality scores with the Wilcoxon signed-rank test.

**Results:**

ChatGPT addressed all (88/88) LUTS/BPE-related questions. For the 88 questions, the recorded F1 score was 0.79 (range: 0–1), precision 0.66 (range: 0–1), recall 0.97 (range: 0–1), and the quality score had a median of 4 (range = 1–5). Examiners had a good level of agreement (ICC = 0.86). We found no statistically significant difference between the scores given by the examiners and the overall quality of the responses (*p* = 0.72).

**Discussion:**

ChatGPT demostrated a potential utility in educating patients about BPE/LUTS, its prognosis, and treatment that helps in the decision-making process. One must exercise prudence when recommending this as the sole information outlet. Additional studies are needed to completely understand the full extent of AI’s efficacy in delivering patient education in urology.

## Introduction

In the midst of growing medical data and reduced accessibility to healthcare professionals, patients are increasingly seeking guidance from search engines and video-streaming platforms [1, 2]. As a result, major urological associations, such as the European Association of Urology (EAU) and the American Urological Association (AUA), have designed online resources that furnish patients with high-quality information and help guide their decision-making process [3, 4]. Nonetheless, the rise of modern technologies has shifted the focus from established institutions to new methods of information garnering such as social media, video tutorials, and artificial intelligence (AI) since they offer more immediate and efficient methods of information retrieval [2, 5, 6].

With more than 1.5 million visits per month and more than 180 million active users, ChatGPT is rapidly becoming the fastest-growing AI language models in the world [7]. This impressive growth can be attributed to its user-friendly chatbot interface, which enables users to pose questions in a conversational style, closely mirroring human interaction [2]. As an increased proportion of physicians and patients continue to explore AI as a tool to further their knowledge or aid the deliverance of healthcare-related services, the need has surfaced to evaluate the accuracy and quality of such technology [1]. A survey, conducted among urologists between April and May 2023, revealed that ~20% had utilized ChatGPT in clinical settings, 56% believed in its potential to aid in clinical decision-making, and more than half suspected that this might be used by their patients for self-management [8].

A limited number of studies, with marked heterogeneity in their results, have been conducted to evaluate the accuracy and reliability of ChatGPT in answering urological queries. In the field of pediatric urology, ChatGPT’s performance showed a 92% accuracy rate when compared to verified resources [9]. Similarly, studies on its ability to educate patients for robotic-assisted prostatectomy found a 79% concordance between source material and ChatGPT’s responses, and even higher rates of accuracy when evaluating only the information provided by the AI chatbot [10]. Conversely, for prostate cancer-related queries, the answers were found to have low performance with an F1 score of 0.426, a precision score of 0.349, a recall score of 0.549, and a general quality score (GQS) levels of 3.62 ± 0.49 [11]. These findings are further corroborated by other studies, evaluating ChatGPT’s answers to multiple urological diseases queries, including BPE, and found a moderate to low quality of responses [12, 13]. Overall, these heterogeneous results suggest that ChatGPT’s effectiveness varies across different urological conditions, potentially performing better in some areas and worse in others.

In light of such conflicting reports about ChatGPT’s quality and accuracy, as well as the lack of current studies investigating its performance in lower urinary tract symptoms (LUTS) suggestive of benign prostate enlargement (BPE) related inquiries, our study aims to evaluate the accuracy and precision of the information provided by ChatGPT’s chatbot (ChatGPT 4.0+) on male LUTS suggestive of BPE compared to two reference resources extracted from the EAU’s patient information portal and the AUA’s patient guide manual.

## Materials and methods

### Information acquisition and data extraction

To start, we scoured the patient information websites from the EAU and AUA in order to identify frequently asked questions and topics of interest. From this, we formulated 88 BPE-centric queries that ranged from concept definition (e.g., “What is BPE?”), symptoms and diagnostics evaluations (e.g., ”What are the normal values of uroflowmetry?”), risk and complications (e.g., What are the risks of BPE?), conservative management and pharmacotherapy (e.g., “What are the most common side effects of α1-blockers in the treatment of BPE?”), and surgical treatment options (e.g., “What minimally invasive procedures are available for BPE?”), see Appendix 1. These questions were then fed to ChatGPT in an independent manner (for each question a new conversation was started) with the response recorded and later compared to the reference material previously mentioned, see Appendix 1.

### Performance metrics

Two examiners independently and in duplicate classified the responses into one of four categories. When examiners found that the ChatGPT’s response was false despite a true version of the statement being present in the source material, they rated it as true negative (TN). If the source material had information not generated by ChatGPT, the examiners classified the response as false negative (FN). Conversely, when ChatGPT’s response was true and verifiable in the source material, examiners categorized it as true positive (TP). If the statement provided by ChatGPT was true according to the current literature but was not present in the reference source, we classified it as false positive (FP). Examiners resolved the discrepancies between the grading by either reaching a mutual consensus or asking a senior specialist.

Once we finished all classifications, we calculated the F1 score, precision, and recall metrics (formula below). We decided to use the F1 score as it is a validated machine-learning metric for the assessment of a model’s accuracy by evaluating the model’s capability of making a correct prediction across a binary class database. The score does this by calculating the harmonic means of both precision and recall measurements on a scale of 0–1, where 0 are all incorrect predictions and 1 represents completely accurate predictions.

$${Precision}=\frac{{TP}}{({TP}+{FP})}{Recall}=\frac{{TP}}{({TP}+{FN})}F1{Score}=2\times \frac{({Precision}\times {Recall})}{({Precision}+{Recall})}$$

### General quality scores (GQS)

We generated a GQS using a 5-point Likert scale (Table 1). The GQS evaluated the quality of the responses generated by ChatGPT. The grading aimed to assess each response’s truthfulness, relevancy, structure, and language. Examiners granted a GQS score of 1 when the information was false or misleading, the text was disorganized or used incomprehensible language, and had zero value to the patient. In contrast, examiners gave a GQS score of 5 when the information was extremely accurate, flawlessly organized, used patient-friendly language, and was totally relevant for the patient. We determined the final GQS score by calculating the mean of the two examiners’ scores for each question.

**Table 1:** General quality score.

General quality score	Description
**1**	- Truthfulness: contains false or misleading information - Relevancy/value: does not answer the question or lacks patient benefit - Structure: disorganized text - Language: uses 90–100% medical jargon or incomprehensible language
**2**	- Truthfulness: information is somewhat accurate but outdated - Relevancy/value: partially addresses the question but offers limited value to patients or >90% of the content is off-topic - Structure: poorly organized text - Language: uses 50–90% medical jargon or unfamiliar language
**3**	- Truthfulness: adequate information but may lack comprehensive details - Relevancy/value: partially addresses the question with some value to patients or 50–90% of the content is off-topic - Structure: semi-organized text - Language: uses <50% medical jargon or unfamiliar language
**4**	- Truthfulness: accurate information - Relevancy/value: fully addresses the question with valuable insights for patients or <50% of the content is off-topic - Structure: well-organized text - Language: uses lay language (7-grade reading level)
**5**	- Truthfulness: extremely accurate information - Relevancy/value: fully addresses the question offering significant value to patients with no irrelevant content - Structure: flawlessly organized text and easy to follow - Language: uses patient-friendly terminology and lay language (7-grade reading level)

We evaluated the level of agreement on the GQS scores between the two examiners using the interclass correlation coefficient (ICC; (2,1)). The difference in the GQS scores between the two examiners was assessed by the Wilcoxon signed-rank test. A *p* value < 0.05 was considered statistically significant. We used SAS version 9.4 for all analyses [14].

## Results

ChatGPT addressed 88 questions across eight categories related to BPE (Table 2). 71.6% of the questions (*n* = 63) focused on BPE management, including conventional surgical interventions (*n* = 27), minimally invasive surgical therapies (MIST, *n* = 21), and pharmacotherapy (*n* = 15) (Table 2). ChatGPT generated responses to all 88 questions with a total of 22,946 words and 1430 sentences. In contrast, the EAU website contained 4914 words and 200 sentences, while the AUA patient guide had 3472 words and 238 sentences. AI-generated responses had almost three times more words than the source material (Table 2). For instance, Table 3 describes a few examples of the responses provided by ChatGPT and the reference materials. Performance metrics of the ChatGPT’s responses fluctuated from 0.5 to 1.0, including F1 score (range = 0.67–1.0), precision scores (range = 0.5–1.0), and recall (range = 0.9–1.0) (Table 2). GQS scores ranged from 3.5 to 5 (Table 2).

**Table 2:** Descriptive statistics and performance metrics by BPE category.

	General concepts	Risk & complications	Symptoms	Testing	Conservative management	Pharmacotherapy	Surgical intervention	MIST
No. of questions	4	6	4	9	2	15	27	21
No. of words ChatGPT	979	1811	950	1870	541	3435	7674	6302
No. of sentences ChatGPT	72	122	63	128	45	247	575	463
No. of words EAU	95	332	260	119	643	781	2159	675
No. of sentences EAU	6	20	24	9	50	58	115	40
No. of words AUA	181	185	323	730	180	214	1139	507
No. of sentences AUA	28	20	31	72	18	17	100	47
F1 score	0.86	1.00	1.00	0.94	1.00	0.75	0.71	0.67
Precision score	0.75	1.00	1.00	0.89	1.00	0.64	0.58	0.50
Recall score	1.00	1.00	1.00	1.00	1.00	0.90	0.94	1.00
GQS Score	4.50	3.50	5.00	4.40	3.50	4.00	4.00	3.80

**Table 3:** Examples of responses provided by ChatGPT and reference materials.

Question	ChatGPT’s response	Reference material
**What is benign prostatic enlargement?**	*You know how some parts of our body can change as we get older? Well, for many men, the prostate gland is one of those parts. The prostate is a small gland that sits below the bladder, kind of like a doughnut around the tube we pee through, called the urethra*. *Benign Prostatic Enlargement, or BPE, is when this gland gets bigger over time. The word ‘benign’ is essential here – it means that this growth isn’t cancerous or harmful in that sense. However, as the prostate grows, it can press on that pee tube (the urethra), making it harder for urine to flow through*. *Here are some things you might notice if your prostate is getting larger:* • *Starting trouble: You might find it a bit hard to start peeing*. • *Stream changes: Your urine flow might be weaker or stop and start*. • *Night visits: You might find yourself getting up to pee more often at night*. • *Rush to go: Sometimes, you may feel a sudden need to pee*. • *Not quite done: After you pee, it might feel like there’s still some left or you might notice a few drops coming out later*. *The good news is, not everyone with a growing prostate will have these issues. And even if you do, we have various ways to help, from medicines to other treatments. If you’re noticing any of these changes, it’s essential to let me know. We can figure out if it’s due to your prostate or something else and then decide on the best steps to take*.	**EAU patient portal:** Benign prostatic enlargement, or BPE, is the medical term for an enlarged prostate, a condition that is common in men over 50. In BPE, the enlarged prostate can block the flow of urine and cause changes to how your bladder stores urine. **AUA patient portal:** Benign Prostatic Hyperplasia (BPH), which is better known as an enlarged prostate, you are not alone. It is a common condition for men as they age. In fact, about half of all men between ages 51 and 60 have BPH. Up to 90 percent of men over age 80 have it.
**What is uroflowmetry?**	*Uroflowmetry is a test we use to see how well you can urinate. Think of it like a speedometer for your urine. It tells us how fast the urine comes out and how much you’re able to release*. *Here’s what you can expect:* *Come Ready: We’ll ask you to arrive with a full bladder, so you’re ready to urinate*. *Private Setup: You’ll be given privacy to urinate into a special toilet or funnel. This device is connected to a computer that does all the measuring*. *The Graph: The computer will make a graph that shows the flow of your urine from start to finish. We look at things like the speed of your flow, how long it takes, and the total amount*. *What We Learn: This graph can give us clues about how well your bladder and the tube you urinate through are working. For example, if the flow is slower than usual, it might mean there’s some blockage or the muscles are weak*. *It’s a straightforward and painless test. The main goal is to better understand what’s happening when you urinate, so we can help with any issues you might be facing*.	**EAU patient portal:** Uroflowmetry is a simple test that electronically records the rate of urine flow. It is easily done in privacy at the hospital or clinic. This test helps your healthcare provider check whether the flow of your urine is blocked. **AUA patient portal:** Uroflowmetry measures how fast urine flows. This is done to diagnose the problem. It may also be done before surgery.
**What are the most common side effects of alpha 1-adrenoceptor antagonists (α1-blockers)?**	*Alpha 1-Adrenoceptor antagonists, or α1-blockers, are generally well-tolerated, but like all medications, they can have side effects. Some of the most common side effects include:* *Orthostatic Hypotension: This is a sudden drop in blood pressure when a person stands up from a sitting or lying position. It can lead to dizziness or feeling lightheaded, and in some cases, fainting*. *Dizziness: Apart from orthostatic hypotension, some people might just feel generally dizzy when taking α1-blockers*. *Fatigue: Some people might feel unusually tired or weak*. *Headache: Some patients report headaches when starting on these medications*. *Nasal Congestion: The medications can cause the blood vessels in the nasal passages to expand, leading to stuffiness or congestion*. *Retrograde Ejaculation: This is when semen goes backward into the bladder during ejaculation instead of out through the penis. It’s harmless and doesn’t affect orgasm, but it can reduce the volume of semen expelled and might interfere with fertility*. *Palpitations: Some people might feel like their heart is beating too hard, too fast, skipping a beat, or fluttering*. *Swelling in the Ankles or Feet: This is due to fluid buildup, also known as peripheral edema*. *Gastrointestinal Symptoms: Some patients might experience nausea, diarrhea, or abdominal discomfort*. *It’s worth noting that while these side effects are possible, they don’t occur in everyone, and some people might experience them more mildly than others. It’s always essential for patients to discuss any side effects or concerns with their healthcare provider. Adjustments in dosage or switching to another medication might be necessary in some cases*.	**EAU patient portal:** Loss of strength, dizziness, and slightly reduced blood pressure, are possible but temporary. It is possible that these medicines can affect sexual function (sex drive and ejaculation). **AUA patient portal:** Side effects may include dizziness, lightheadedness, fatigue and difficulty ejaculating. Men with moderate to severe BPH and men who are bothered by their symptoms are good candidates. Alpha blockers are not a good choice if you plan to have cataract surgery soon.

**Fig. 1:**
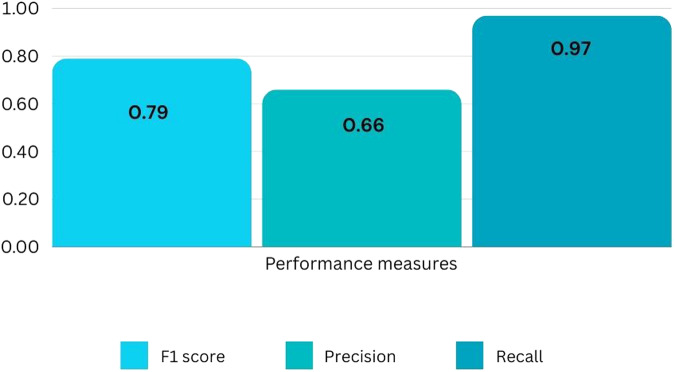
ChatGPT’s performance metrics. F1 score, precision, and recall of ChatGPT’s responses on male lower urinary tract symtpoms suggestive of benign prostate enlargement compared to the European Association of Urology and the American Urological Association patient resources.

The overall performance metrics showed an F1 score of 0.79, a precision score of 0.66, and a recall score of 0.97 (Fig. 1). The GQS scores from both examiners had a median of 4 (range = 1–5). When compared, the examiners found no statistically significant difference between the scores they assigned to the overall quality of the responses (*p* = 0.72), and they determined a good level of agreement between them, with an ICC of 0.86.

## Discussion

The integration of AI into medical practice is an evolving and innovative initiative that has gained attention for its potential to enhance information availability, guide decision-making processes, and optimize executive operations within healthcare [1, 15]. Notably, ChatGPT has already demonstrated its utility in other medical domains by successfully accomplishing tasks traditionally performed by physicians, such as test-taking, medical record documentation, and scientific literature production [15–18]. Nonetheless, its competency in delivering accurate and patient-friendly information remains controversial. Our study sought to assess the precision, accuracy, and quality of ChatGPT’s chatbot-generated responses to common patient queries regarding BPE definitions, symptomatology, diagnostic testing, risks, and treatments.

Our results indicated that ChatGPT was able to respond to all 88 queries. Performance metrics, such as F1 scores, precision, and recall, consistently remained above 0.5 suggesting a consistency level of over 50% between responses and source materials. In terms of content quality, the overall GQS score was 4, with 86% of queries attaining the maximum score of 5. However, it is important to note the word count difference between the chatbot responses and the source materials. We observed that despite the accuracy of the content, the information provided by ChatGPT could be excessive, producing three times more words than the original materials, and not entirely relevant to the patient’s needs. For instance, when asked about alpha-blockers in the treatment of BPE, the AI’s response included extensive explanations of its usage in hypertension which, while accurate, may not have been directly pertinent to the patient’s query regarding BPE treatment.

The accuracy and quality of the model fluctuated greatly depending on the inquired topic. It excelled in areas regarding BPE concept, symptoms, and diagnostics, with F1 scores ranging from 0.86 to 1 and GQS scores above 4.4. However, it did not perform as well when addressing topics related to MIST, with the lowest precision score of 0.67 recorded and GQS scores that are notably lower. Such variation is anticipated since emergent technologies often have limited or outdated data for AI systems like ChatGPT to extract from.

Our findings align closely with what has been documented in existing literature, where significant accuracy heterogeneity has been found not only across researchers but also within subtopics of a given paper, mainly due to prompt composition and ability to validate the pertinence of the answers [19, 20]. This is evident in the exploration of ChatGPT’s application within various areas of urology, including urologic oncology, sexual health, and pediatric urology [9, 13, 21]. Although there is a lack of consensus regarding the overall accuracy of ChatGPT’s responses, the majority of studies agree that its performance varies depending on the type of question and what it pertains to [19, 22]. It was generally observed that the most accurate responses had to do with quality of life or information-based queries whereas decision-making questions lacked accuracy and consistency [23].

In addition to all the potential applications in the medical field, it is crucial to consider the legal implications associated with the deployment of such technologies. There are legal gaps related to liability, accountability, and data protection policies that should be addressed before their integration into our practice [24]. Without ensuring accountability for potential medical outcomes and confidentiality breaches, their progress in our field will be in vain. Nevertheless, we recognize the potential of all AI technologies in aiding a wide range of health-related fields, such as radiological and histological analysis, prediction models, and prognostic assistance [23].

As an academic exercise, we asked ChatGPT to list its potential contributions to the medical field. Among ChatGPT’s responses, the integration of AI into wearable devices for real-time monitoring and the application of image/pattern recognition are particularly promising and achievable. However, it is essential to remember that the speculation on future developments and the realization of actual advancements will depend solely on ongoing research, technological progress, and the ethical considerations surrounding them.

One of the largest limitations of the study was the lack of validated questionnaires to evaluate ChatGPT’s response quality, as well as the scarce information regarding suitable metrics to assess AI. We recognize that the 5-point Likert scale we employed for assessment introduces a degree of subjectivity into the ratings, as examiners might differ in valuations of specific criteria. Furthermore, it is important to acknowledge that the version of ChatGPT used had access to information only up until April 2023. Therefore, any further improvements to the AI model or additions to BPE data could not be captured in our current findings. Overall, this study adds to the ongoing discussion on ChatGPT’s performance highlighting its accuracy and reliability. Nonetheless, more studies including patient evaluation of the information delivered by ChatGPT compared to reference material and with a broader inclusion of pathologies need to be conducted to truly generalize the usage of this tool across the field of urology.

## Conclusion

ChatGPT, as an AI-powered chatbot, demonstrates a potential utility for educating patients about BPE, its prognostic outcomes, and management strategies that aid the decision-making process. Nonetheless, one should be cautious when advising patients to use this as the exclusive source of information, particularly when considering minimally invasive procedures. As novel technologies continue to progress and become more integrated within healthcare settings, we anticipate they will become significant conduits for information acquisition. Additional studies are needed to completely understand the full extent of AI’s efficacy in delivering patient education.

## Supplementary information

Appendix 1

## Data Availability

The dataset analyzed during the current study is available in the supplementary material (Appendix 1).
