# Supplementary material for: Can ChatGPT provide high-quality patient information on male lower urinary tract symptoms suggestive of benign prostate enlargement?
Source: Prostate Cancer Prostatic Dis. 2024 Jun 13;28(1):167–72. doi: 10.1038/s41391-024-00847-7 (PMC11860216; doi:10.1038/s41391-024-00847-7)
Supplement: Supplementary file 1 — Appendix 1 [file 41391_2024_847_MOESM1_ESM.docx]

**Appendix 1. 88 BPE-centric queries entered to ChatGPT 4**

| **Topic of interest** | **Question** | **ChatGPT’s response** | **Reference material** |
| --- | --- | --- | --- |
| About BPE | 1. What is the prostate? | The prostate is a small gland in your body that's part of the male reproductive system. It's located just below your bladder and near the rectum. Imagine it like a walnut in size and shape. It plays a big role in your urinary and sexual health.  One of its main jobs is to help make semen, the fluid that carries sperm. The prostate produces a special fluid that mixes with sperm from your testicles and other fluids to form semen. This fluid is important because it helps keep the sperm healthy and able to fertilize an egg.  As men get older, the prostate can grow larger. This is common and usually not dangerous, but it can sometimes cause problems with urinating, because the prostate surrounds part of the tube that urine flows through. If you notice any changes or difficulties in urinating, it's important to let a doctor know.  Also, there's something called prostate cancer, which is one of the more common cancers in men. Regular check-ups are important because if found early, it's often treatable.  Lastly, there's a condition called prostatitis, where the prostate becomes inflamed or infected. It can cause discomfort and urinary issues.  Remember, taking care of your prostate is an important part of your overall health, especially as you get older. If you have any concerns or symptoms, don't hesitate to talk to your doctor about them. | *EAU patient portal:*  The prostate is a small gland that forms part of a man’s reproductive system. It surrounds the urethra tube that empties urine from the bladder.  *AUA patient portal:*  The prostate is part of the male reproductive system. The prostate’s main job is to make fluid for semen. It is about the size of a walnut and weighs an ounce or so. It sits below the bladder and in front of the rectum. It goes around a tube called the urethra. The urethra carries urine from the  bladder and out through the penis. |
|  | 2. What are lower urinary tract symptoms? | LUTS, or Lower Urinary Tract Symptoms, is a term we use to describe a range of symptoms you might experience when it comes to urinating. Think of your urinary system as a plumbing system: sometimes there can be issues with how it stores or releases water (in this case, urine). Here's a breakdown:  Storage Problems:  You feel like you need to go to the toilet more often than you used to.  You wake up in the middle of the night needing to pee.  Sometimes, you feel a sudden, strong need to urinate and can't wait.  Occasionally, this strong need may lead to leakage before you reach the toilet.  Emptying Problems:  When you try to pee, the stream feels weak or slow.  You might find it hard to get started, even when you feel you need to go.  Sometimes, you might have to push a bit to get the urine out.  The flow might stop and start instead of being continuous.  After you finish, it might feel like there's still some urine left in the bladder.  You might also notice a few drops of urine leaking after you think you're done.  Now, many people, especially as they get older, experience some of these symptoms. They can be caused by a variety of factors, from an enlarged prostate in men to bladder infections or other conditions. It's essential to share with me which of these symptoms you're experiencing, so we can pinpoint the cause and work on the best treatment for you. | *EAU patient portal:*  Lower urinary tract symptoms. A term used for the symptoms caused by BPE which can also point to other diseases affecting the urinary tract (see also Urinary tract).  *AUA patient portal:*  As the prostate enlarges, it presses against the urethra. The bladder wall becomes thicker. Over time the bladder may weaken and lose the ability to empty fully. Urine then remains in the bladder. These problems cause many of the lower urinary tract symptoms (LUTS) of BPH. How BPH causes symptoms may help your health care team determine if and what type of treatment to suggest. |
|  | 3. What is benign prostatic enlargement? | You know how some parts of our body can change as we get older? Well, for many men, the prostate gland is one of those parts. The prostate is a small gland that sits below the bladder, kind of like a doughnut around the tube we pee through, called the urethra.  Benign Prostatic Enlargement, or BPE, is when this gland gets bigger over time. The word 'benign' is essential here – it means that this growth isn't cancerous or harmful in that sense. However, as the prostate grows, it can press on that pee tube (the urethra), making it harder for urine to flow through.  Here are some things you might notice if your prostate is getting larger:  Starting trouble: You might find it a bit hard to start peeing.  Stream changes: Your urine flow might be weaker or stop and start.  Night visits: You might find yourself getting up to pee more often at night.  Rush to go: Sometimes, you may feel a sudden need to pee.  Not quite done: After you pee, it might feel like there's still some left or you might notice a few drops coming out later.  The good news is, not everyone with a growing prostate will have these issues. And even if you do, we have various ways to help, from medicines to other treatments.  If you're noticing any of these changes, it's essential to let me know. We can figure out if it's due to your prostate or something else and then decide on the best steps to take. | *EAU patient portal:*  Benign prostatic enlargement, or BPE, is the medical term for an enlarged prostate, a condition that is common in men over 50. In BPE, the enlarged prostate can block the flow of urine and cause changes to how your bladder stores urine.  *AUA patient portal:*  Benign Prostatic Hyperplasia (BPH), which is better known as an enlarged prostate, you are not alone. It is a common condition for men as they age. In fact, about half of all men between ages 51 and 60 have BPH. Up to 90 percent of men over age 80 have it. |
|  | 4. What is bladder outlet obstruction? | Imagine your bladder as a water balloon with a nozzle at the bottom. When you're ready to empty the balloon, you'd open the nozzle, and water flows out. Now, think of your bladder trying to release urine through a tube called the urethra. Bladder Outlet Obstruction, or BOO for short, is like having something partially or fully blocking that nozzle. It means that something is getting in the way of the urine flowing out smoothly.  So, what could be causing this block? There are a few possibilities:  In men, a growing prostate: This is a common cause where the prostate gland, sitting around the urine tube, gets bigger and squeezes the tube.  Narrowed tube: Sometimes, the tube (urethra) can become narrow or scarred, making it harder for urine to flow.  Blockages: There could be little stones or even growths that are getting in the way.  Muscle or nerve issues: Sometimes, the problem might be with how the bladder's muscles open or how the nerves tell them to act.  If you have this blockage, you might:  Find it hard to start peeing or have a weak flow.  Feel like you haven't completely emptied your bladder after going.  Need to go more often or urgently.  Wake up multiple times at night to pee.  It's crucial to let me know if you're facing any of these issues. We can check to see what's causing the block and talk about how to make things better. | No information available in EAU and AUA patient portals |
| Symptoms of BPE | 5. What are the symptoms of benign prostatic enlargement? | Imagine your prostate gland is like a small doughnut located near the bladder. When you get older, this 'doughnut' can sometimes get bigger. This is usually not cancer, but it can press against the tube where urine flows (the urethra), which might cause some changes or problems with how you pee.  Here are some things you might notice if your prostate is enlarged:  Needing to Pee More Often: You might find that you need to go to the bathroom more often, especially at night.  Urgent Feeling to Pee: Sometimes, you might suddenly feel a very strong need to pee.  Trouble Getting Started: When you try to pee, it might take a while to get started, or you might have to push harder.  Weak Stream: The flow of your pee might be weaker than usual, or it might stop and start.  Dribbling After You Finish: After you think you’re done peeing, you might find a few drops still coming out.  Feeling Like You Haven’t Emptied Your Bladder: Even after you pee, you might feel like your bladder isn’t completely empty.  If you’re experiencing any of these things, it’s a good idea to talk to your doctor. These symptoms are common as men get older, and there are ways to manage them. Your doctor might suggest some tests to see how your prostate is doing and discuss different options to help you feel better. Remember, dealing with these symptoms early on can make a big difference! | *EAU patient portal:*  BPE can result in a range of symptoms including:  A delay in starting and/or finishing urinating  Having to strain to urinate  A weak stream of urine  Stopping and starting while urinating  Spraying urine while going to the toilet, or the urinary stream splitting  The feeling that you cannot fully empty your bladder  Needing to urinate more often than is normal and/or more urgently  Waking up at night to urinate  Finding it difficult to hold or control your bladder  Accidentally leaking urine (urinary incontinence)  In some men, the symptoms are mild and do not need treatment. In others, they can be troublesome and can benefit from treatment.  *AUA patient portal:*  When the prostate is enlarged, it can bother or block the bladder. Needing to pass urine often is a common symptom of BPH. This might include the need to pass urine as often as every one to two hours, mainly at night.  Other symptoms of BPH are listed below:  • Incomplete emptying: the feeling your bladder is full, even after passing urine.  • Frequency: the need to pass urine often, about every one to two hours.  • Intermittency: the need to stop and start several times when passing urine.  • Urgency: feeling the urgent need to pass urine as if you can’t wait.  • Weak stream: a weak urine flow.  • Straining: trouble starting to pass urine or the need to push or strain to pass urine.  • Nocturia: the need to wake up at night more than two times to pass urine. |
|  | 6. What is the International Prostate Symptom Score? | The International Prostate Symptom Score, or IPSS, is like a quick survey we use to understand any pee-related problems you might be having. It's particularly helpful for us to get a clear picture of how you're feeling, especially if we suspect issues with your prostate, which is a common gland in men that can sometimes grow and cause urinary changes.  Here's what we'll ask you:  About Your Symptoms:  Emptying: Do you ever feel like your bladder isn't entirely empty after going to the bathroom?  Frequency: How often do you feel like going back to pee shortly after you just went?  Nighttime: How often are you waking up at night to use the bathroom?  Flow: Do you notice your pee stream is weaker than before?  Holding it in: Is it hard for you to hold in your pee when you feel the urge?  Starting: Do you have to push or strain to start peeing?  Stop-and-Go: When peeing, do you often stop and start?  You'll mark your answers based on how often you experience each thing, from "Not at all" to "Almost always."  How You Feel About It:  We also want to know how you'd feel if these pee problems stayed with you forever. Would you be okay with it, or would it make you pretty upset?  By answering these questions, you help me understand what's going on and how best to help you. It's also a great way for us to see if any treatments are working or if things are changing over time. | *EAU patient portal:*  No information available  *AUA patient portal:*  If you have any urinary changes or symptoms, your doctor may talk to you about the BPH Symptom Score Index. The American Urological Association (AUA) developed this test to assess urinary symptoms.  This is often the first step to diagnose BPH. The score can rate BPH as being mild to severe. You and your health care provider may talk about your results and your medical history. This is sometimes also called the International Prostate Symptom Score (IPSS) |
|  | 7. How would you classify the International Prostate Symptom Score? | The International Prostate Symptom Score (IPSS) is classified into severity categories based on the total score obtained from the seven symptom questions (excluding the quality of life question). Here's how the scores are typically classified:  Mild (0-7):  This range indicates that the urinary symptoms are mild and may not be overly bothersome to the patient.  Moderate (8-19):  Patients in this range are experiencing moderate symptoms. They might find their symptoms more noticeable and occasionally bothersome.  Severe (20-35):  This is the highest range, indicating severe urinary symptoms that can be quite disruptive to daily life and activities.  The eighth question about quality of life isn't used in this classification, but it provides valuable context about how the patient feels about their symptoms.  When a patient completes the IPSS, a healthcare provider can use the classification to guide treatment decisions, with more aggressive treatments often considered for higher scores (especially if the quality of life is impacted). However, individual patient preferences and clinical judgment are also essential in guiding management. | *EAU patient portal:*  No information available  *AUA patient portal:*  Total score: 0–7 mildly symptomatic; 8–19 moderately symptomatic; 20-35 severely symptomatic. |
|  | 8. What is a bladder diary? | Think of a bladder diary as a personal journal for your bladder's activities. It's a way for us to get a detailed look at what's going on with your bathroom habits over a couple of days. By tracking specifics, we can better understand any issues you might be facing and decide on the best way to help.  So, what would you jot down in this diary?  When You Go: Each time you head to the bathroom, write down the time and date.  How Much You Go: If possible, measure how much you pee using a special container (I can provide you with one) and note down the amount.  Urgent Needs: If you felt a sudden, strong need to pee, make a note of it.  Any Leaks: If you have moments where urine leaks out without you wanting it to, write down when it happens, how much, and maybe what you were doing (like sneezing or laughing).  Drinks: Keep track of what you drink, how much, and when. This includes everything—water, tea, coffee, sodas, and so on.  Other Details: If you feel any pain or discomfort while peeing or notice anything else unusual, jot that down too.  Medicines: If you're taking any medications, especially those that might affect how often you pee, list them.  Protective Products: If you use any pads or protective underwear, note down which ones and if they get wet.  It might feel like a bit of homework, but it's just for a short while, usually 1 to 3 days. The information you provide is super helpful for me to see patterns and get a clear picture of what's happening. Together, we can then work on a plan to improve your comfort and health. | *EAU patient portal:*  Your doctor may ask you to keep a bladder diary. This can help diagnose urinary problems, including overactive bladder and benign prostatic enlargement. The bladder diary is important because it helps your doctor understand your symptoms better.  The bladder diary will note the number of times you urinated, the amount of urine passed, the pad weight if you are using pads, and times when you had incontinence or urgency. This is typically done over 24 hours and for 3 consecutive days to help identify the problem.  You will keep track of how much you drink, how often you go to the toilet to pass urine, and how much you produce by measuring the amount of urine at home with the help of a measuring jug. You may use a stopwatch to record the time it took you to urinate. Note down the amount of urine (in millilitres) and the time (in seconds).  *AUA patient portal:*  No information available |
| Risks and complications of BPE | 9. What are the risks of benign prostatic enlargement? | Benign Prostatic Enlargement (BPE) means that the prostate gland, which is a small gland below the bladder in men, has grown bigger than usual. It's not cancerous, but as it grows, it can press against the tube through which you pee, causing a few problems. Let's talk about the risks or complications this can lead to:  Infections: Because the urine might not flow out as smoothly, there's a chance for bacteria to grow, leading to urinary infections.  Stones in the Bladder: If the bladder doesn't empty out entirely, some minerals can form stones over time, just like how kidney stones form.  Blockage: The growing prostate can block the urine flow, making it hard to pee.  Weak Bladder: Over time, the bladder might have to work harder to push out urine, which can make its walls weaker.  Kidney Issues: If the bladder pressure gets too high, it can affect the kidneys and potentially harm them.  Sudden Inability to Pee: In some cases, you might find you can't pee at all suddenly. This can be painful and needs immediate attention.  Always Having Some Urine Left: Even after you pee, there might be some urine left in the bladder consistently.  Blood in Pee: Sometimes, you might notice a pinkish tint or blood in your urine.  Issues in the Bedroom: Some men find they have problems with getting or maintaining an erection or with ejaculation.  Bathroom Problems: You might find yourself needing to go more often, especially at night, or feeling a sudden strong urge to pee.  It's important to remember that while these are potential risks, not everyone with a growing prostate will face all of them. It varies from person to person. By keeping regular check-ups and letting me know of any changes or symptoms, we can manage these risks and keep you feeling your best. | *EAU patient portal:*  BPE is not cancer. In some cases, it can lead to serious complications such as kidney disease or the inability to urinate. You should always contact your doctor if you have any difficulty urinating.  *AUA patient portal:*  No information available |
|  | 10. What are the causes of benign prostate enlargement? | Think of your prostate as a small gland located below your bladder. As men get older, this gland can grow bigger, a condition we often call benign prostatic hyperplasia or BPH. Now, you might wonder why this happens. Here's what we know:  Getting Older: Just as hair turns gray with age, the prostate tends to grow. It's just a part of aging for many men.  Hormone Changes: As men age, there's a shift in the balance of male hormones. This change might cause the prostate to grow.  Family History: If your dad or grandpa had an enlarged prostate, you might be more likely to have it too. It seems to run in families.  Where You're From: Men from certain parts of the world, like Western countries, seem to have a higher chance of the prostate growing larger.  Lifestyle & Diet: Being significantly overweight or eating a lot of fatty foods might increase the risk. On the other hand, a diet rich in fruits and veggies might help keep the prostate from growing too much.  Other Health Conditions: If you have conditions like diabetes, high blood pressure, or heart disease, you might be at a higher risk.  Medications: Some medicines, especially those related to male hormones, can make the prostate grow.  It's essential to know that an enlarged prostate is not cancer. It's a benign condition, which means it's not harmful by itself. But, it can cause some bothersome symptoms like trouble with urination. If you're facing such issues, we're here to help and guide you through it. | *EAU patient portal:*  No information available  *AUA patient portal:*  The causes of BPH are not clear. It mainly occurs in older men. Hormone changes are thought to play a role.  Hormones from the testis may be the main factor. For example, as men age, the amount of active testosterone in the blood declines. Estrogen levels stay the same.  BPH may occur when these hormone changes trigger prostate cell growth. Another theory is about the role of dihydrotestosterone (DHT). This male hormone supports prostate development. Some studies show that older men have higher levels of DHT. Testosterone levels go down. |
|  | 11. Who is at risk for developing benign prostatic enlargement? | Hello! I'd like to talk to you about a condition called benign prostatic enlargement, or BPE. Some people also call it BPH. It's where the prostate gland grows larger over time, which can sometimes affect how you urinate.  Who's at Risk?  Age: As men get older, the prostate can grow in size. This is a natural process, and many men over 60 experience some growth.  Family: If your father, brother, or other close male relatives had this condition, you might have a higher chance of experiencing it too.  Background: Men from different parts of the world have different chances of getting BPE. For example, men of African or Caribbean background may have a slightly higher risk, while men of Asian background may have a lower risk.  Health: Some health issues like being overweight, having diabetes, or heart problems can increase the chance of getting BPE.  Medicines: Some medicines, like certain cold or allergy pills, can make the symptoms of BPE more noticeable. They don't cause the growth, but they can make the symptoms feel worse.  What does this mean for you?  Even if you have one or more of these risk factors, it doesn't mean you'll definitely have BPE. And if you don't have any, it doesn't mean you're completely safe. It's just like any other health condition where some people are more likely to get it than others.  If you ever notice changes in how you urinate, like going more often or feeling like you can't empty your bladder completely, it's a good idea to let your doctor know. They can check if it's related to BPE and suggest ways to help.  Remember, it's always a good idea to have regular check-ups and chat about any concerns with your doctor! | *EAU patient portal:*  No information available  *AUA patient portal:*  • Men over the age of 50 as the risk for BPH rises with age  • Men whose fathers had BPH  • Men who are overweight or obese  • Men who don’t stay active  • Some men with erectile dysfunction (ED) |
|  | 12. How does benign prostatic enlargement affect my daily life? | Benign prostatic enlargement, often called BPH, is a common condition as men get older. It means that your prostate gland, which is near your bladder, has gotten larger. This enlargement is not cancerous, but it can affect how you urinate.  Here's what you might notice:  Urination Changes: You may feel like you need to urinate more often, both during the day and at night. Starting to urinate might take a bit longer, and the stream might not be as strong as it used to be. After you finish, you might still feel like your bladder isn’t completely empty.  Sleep Disruptions: Because you might need to go to the bathroom more at night, your sleep could be interrupted. This can make you feel tired during the day.  Daily Activities: These changes can affect your daily life. You might find it hard to be far from a bathroom, which can affect work or social activities.  Other Effects: Sometimes, BPH can affect sexual function or lead to feelings of stress or frustration. It's not uncommon to feel anxious or down about these changes.  Physical Activity: You might feel like you need to limit activities to stay close to a bathroom, but it's important to stay active for your overall health.  The good news is that there are treatments available. These might include medications or lifestyle changes, and they can really help manage these symptoms. We can work together to find the best approach for you.  Remember, you're not alone in this. Many men experience these changes as they get older. It's important to keep an open dialogue about how you’re feeling and any symptoms you’re experiencing. This way, we can ensure you get the best possible care. | *EAU patient portal:*  The symptoms of BPE, such as needing to urinate urgently or very often, can be difficult to deal with. You may be worried about carrying out your usual social activities due to fear that there may not be a toilet nearby. It is important that you do not avoid social activities that you enjoy. Instead, go to your doctor, who will be able to give you advice and discuss possible treatment options that can help you manage your symptoms.  *AUA patient portal:*  No information available |
|  | 13. How does benign prostatic enlargement affect my sexual ability? | Benign Prostatic Enlargement (BPE), which is a common condition where the prostate gland gets larger as men get older, can sometimes affect your sexual health. Here's how:  Trouble with Erections: Sometimes, BPE can make it harder to get or keep an erection. This isn't the same for everyone, and it's not always clear why it happens. Some medicines for BPE can also make this problem worse.  Changes in Ejaculation: With BPE, you might notice differences when you ejaculate. It could feel like there's less force or less fluid, or it might feel like not everything comes out. In rare cases, the fluid might go into the bladder instead.  Feeling Less Interested in Sex: BPE doesn't directly make you less interested in sex, but if you're worried about your symptoms or if they're bothering you a lot, that can make you feel less like having sex. Also, some treatments for BPE might change your hormone levels, which can affect how much you're interested in sex.  Pain or Discomfort: A few men might feel pain or discomfort when they ejaculate, which can affect how enjoyable sex is.  Remember, how much BPE affects your sex life can really vary from person to person. Some men might have a lot of trouble, while others might not notice much difference.  If you're having any of these problems, it's a good idea to talk to your doctor. They can help figure out if BPE is the cause or if something else is going on. They can also talk about treatments that can help both your prostate and your sex life. | *EAU patient portal:*  BPE symptoms can have a negative effect on your personal relationships and sex life. It can be difficult to feel confident when you do not always feel in control of your body. Episodes of leaking or needing to find a toilet quickly can be embarrassing and lower your self-esteem.  Side-effects of drug treatments, such as lack of sexual drive or erectile dysfunction, can add to these feelings.  These changes can be difficult to deal with and some men can suffer from depression as a result. That is why the effect of BPE on a person’s quality of life should not be underestimated. Living with BPE can be challenging for yourself and your partner, so it’s very important that you talk together and discuss the best way to cope with this condition. Together with your partner and your doctor, you can identify what is important in both your everyday life and your sex life and choose the best treatment option, to make it easier for you to live with BPE.  *AUA patient portal:*  No information available |
|  | 14. Can a patient prevent benign prostatic enlargement? | Benign Prostatic Enlargement (BPE) or Benign Prostatic Hyperplasia (BPH) is a common condition in aging men. While its exact cause is not completely understood, it's believed to be influenced by factors like aging and the male hormone testosterone. As such, BPE is often considered a natural part of aging for many men.  While you cannot entirely prevent BPE, some lifestyle changes and choices might help reduce the risk or delay its onset:  Diet: A balanced diet rich in fruits, vegetables, and whole grains can promote overall health and might be linked to a reduced risk of BPE. Some studies suggest that foods rich in beta-sitosterol (like some nuts and seeds) can alleviate BPE symptoms.  Limit Caffeine and Alcohol: Reducing intake of beverages that can irritate the bladder or increase urine production might help manage symptoms.  Regular Exercise: Regular physical activity can help maintain a healthy prostate and reduce the risk of BPE.  Maintain a Healthy Weight: Obesity might be linked to an increased risk of BPE and related urinary symptoms. Maintaining a healthy weight through diet and exercise can be beneficial.  Regular Check-ups: Regular medical check-ups, especially as one gets older, can help in early detection of prostate issues. Early management can prevent complications and improve quality of life.  Medication Awareness: Some medications, like decongestants and antihistamines, can worsen BPE symptoms. It's essential to discuss any new drugs with your healthcare provider.  Limit Fluid Intake Before Bed: Reducing fluid intake in the evening can decrease the need to get up during the night to urinate, a common symptom of BPE.  Kegel Exercises: Strengthening the pelvic floor muscles can help control urination.  Bladder Training: This involves training your bladder to hold urine longer. It requires increasing the intervals between using the bathroom over time, helping increase bladder capacity and control.  Avoid Tobacco: Smoking and tobacco use might be associated with a higher risk of BPE and other urinary symptoms.  Soy-Based Diet: Some evidence suggests that diets high in soy might be associated with a lower risk of BPE, possibly due to the estrogen-like effects of compounds found in soy.  However, it's important to note that while these strategies might help manage or reduce the symptoms of BPE, they don't directly prevent the enlargement of the prostate. Genetics, hormonal changes, and aging are primary factors influencing BPE, and they cannot be modified. Always consult with a healthcare professional before making significant changes to your lifestyle or health regimen. | *EAU patient portal:*  There are no known ways of preventing BPE, as its causes are unknown. BPE is more common in older men, so it is thought that advancing age is an important factor.  It is always a good idea to maintain a healthy lifestyle, which includes staying active and eating a balanced diet.  *AUA patient portal:*  There is no sure way to prevent BPH. Still, losing weight and eating a well-balanced diet, rich in fruits and vegetables, may help. Too much body fat may increase hormone levels and other factors in the blood and stimulate the growth of prostate cells. Staying active also helps control weight and hormone levels. |
| Tests and diagnostics | 15. What tests are available for benign prostatic enlargement? | Benign Prostatic Enlargement (BPE) is when the prostate gland, found below the bladder in men, grows larger than usual. If we suspect you have this condition, we can use several tests to get a clearer picture:  Physical Check: I'll use a gloved finger to feel the prostate through the rectum. It helps check its size and shape.  PSA Blood Test: We take a small blood sample to measure PSA levels. This can tell us more about the prostate's health.  Urine Test: This helps us see if there's an infection or any blood in the urine.  Flow Test: You'll urinate into a special device that measures how fast the urine flows.  Leftover Urine Check: After you pee, we might use an ultrasound or a small tube to check if any urine remains in the bladder.  Camera Test (Cystoscopy): A thin tube with a camera is inserted into the urethra to take a look inside the bladder and at the prostate.  Pressure Test: We measure the strength of the bladder when you pee, to see if the prostate is causing any blockage.  Bladder Function Test: Checks how well your bladder stores and releases urine.  X-ray with Dye (IVP): This helps us see the pathway of urine by injecting a dye and taking X-rays.  Ultrasound: A painless scan that gives us images of the prostate, bladder, and kidneys.  X-ray during Peeing: We take X-rays while your bladder is full and as you pee.  Questionnaire: You'll answer some questions about your symptoms. This helps us understand how much they're affecting your day-to-day life.  Remember, not everyone needs all these tests. Depending on your symptoms and initial results, we'll decide which ones are right for you. The goal is to understand what's going on and then decide on the best plan to help you. | *EAU patient portal:*  No information available  *AUA patient portal:*  These tests are done to measure how well you release urine . This shows the doctor if the urethra is blocked or obstructed . There are several types:  • Urinalysis tests your urine sample to check for important things such as blood, signs of infection, glucose (sugar), protein and other factors that can tell your urologist the cause of your symptoms. Urine tests are also used to screen for bladder cancer. If you have blood in your urine, pain or burning when you pass urine, or you cannot pass urine, it is important to see your doctor right away.  • Post-void residual volume (PVR) measures urine left in the bladder after passing urine. This is done to diagnose the problem . It may also be done before surgery.  • Uroflowmetry measures how fast urine flows. This is done to diagnose the problem. It may also be done before surgery.  • Urodynamic pressure flow study tests pressure in the bladder during urination.  These tests are done to see the size and shape of the prostate . Some BPH scans include:  • Ultrasounds look inside the body and see the size and shape of the prostate.  • Cystoscopy is an exam used to look at the urethra or bladder with a scope.  • Magnetic resonance imaging (MRI) and computed tomography (CT) are more detailed scans. These are done if surgery is necessary to reopen the flow of urine. These scans provide a very clear image of the prostate and surrounding area . It shows exactly how and where the prostate is enlarged.  Blood Tests  If cancer is suspected, blood tests may be done.  • Prostate-specific antigen (PSA) blood tests are used to screen for prostate cancer. The PSA blood test checks the level of PSA, a protein made only by the prostate gland. This blood test can be done in a lab, hospital or a provider's office. Avoid sexual activity several days prior to the test, as this may artificially increase the PSA reading. When the prostate is healthy, very little PSA is found in the blood. A rapid rise in PSA may be a sign that something is wrong. A benign (non-cancer) enlargement of the prostate can cause a rise in PSA levels, as can inflammation of the prostate (prostatitis). The most serious cause of a rise in PSA is cancer. |
|  | 16. What is a digital rectal exam? | It's a routine procedure and is quite quick, usually taking only a few minutes. This exam helps us check the health of some internal organs, including the prostate gland for men. Here's what will happen:  Preparation: We'll ask you to change into a gown for your comfort and privacy. You'll be positioned in a way that's best for the exam, typically lying on your side with your knees bent towards your chest. This position helps us to perform the exam effectively.  The Exam Itself: I'll wear gloves and use a lubricant to make the exam as comfortable as possible. I'll gently insert a finger into your rectum. This allows me to feel the organs inside, such as the prostate gland in men. The purpose is to check for anything unusual, like lumps or swelling. The exam is brief and should not be painful, though it might feel a bit uncomfortable or strange.  After the Exam: If I notice anything that needs further evaluation, I'll discuss it with you and we can plan the next steps, like additional tests. If everything feels normal, that's great news, and we'll continue with any other parts of your physical exam as needed.  Remember, this exam is an important part of taking care of your health. It helps us catch any potential issues early, when they're usually more manageable. I'll make sure to do everything I can to keep you comfortable during the exam, and please let me know if you have any questions or concerns. | *EAU patient portal:*  No information available  *AUA patient portal:*  A digital rectal exam (DRE) is often the next step.  During a DRE, you lie on your side or bend over. The doctor inserts a gloved, lubricated finger into your rectum to feel the back wall of the prostate gland. The health care provider is looking for enlargement, tenderness, lumps or hard spots. This 10-15 second exam is an important way to find problems. |
|  | 17. What is the normal prostate size/volume? | The size of the prostate gland can vary among individuals, but there is a general range considered "normal" for different age groups. It's important to note that "normal" doesn't necessarily mean "healthy" in every case, as some men with a prostate size within the normal range can still experience symptoms of Benign Prostatic Enlargement (BPE) or other prostate conditions.  In general:  Young Adults: In men in their 20s, the prostate is typically about the size of a walnut and weighs approximately 15 to 20 grams.  Middle-aged Men: As men age, particularly after the age of 40, it's not uncommon for the prostate to begin enlargening. By the age of 60, many men have prostates that are larger than the typical size for young adults, but this doesn't always result in symptoms.  Older Men: For men over 60, the prostate can weigh 30 grams or more. Even larger sizes (40 grams to 100 grams or more) can be observed in men with significant BPE.  The size or volume of the prostate can be measured in various ways:  Digital Rectal Examination (DRE): This provides a rough estimate of prostate size.  Transrectal Ultrasound (TRUS): This imaging test provides more accurate measurements of the prostate's dimensions, from which its volume can be calculated. A common formula used to estimate prostate volume based on TRUS measurements is the ellipsoid formula: height x width x length x 0.52.  It's crucial to remember that prostate size alone doesn't always correlate directly with symptom severity. Some men with larger prostates may have minimal symptoms, while others with relatively smaller prostates may experience significant urinary symptoms. | *EAU patient portal:*  A healthy prostate is between 15 and 25 mL, which is about the same size as a golf ball.  *AUA patient portal:*  The prostate is usually the size of a walnut or golf ball in adult men. |
|  | 18. How can the prostate size/volume be measured? | To measure the size of your prostate, which is a small gland located below your bladder, we have several methods:  Physical Check (DRE): This is when I use a gloved finger to feel the prostate by gently pressing against the wall of the rectum. It gives us a basic idea of the prostate's size and shape.  Ultrasound from Inside (TRUS): This involves placing a small ultrasound device into the rectum to get images of the prostate. It's a bit like the ultrasounds pregnant women get, but focused on the prostate. We can get a good idea of its size using this method.  MRI: It's a type of scan that gives us detailed pictures of the prostate. It's especially useful if we need a clear view of both the prostate and the surrounding area.  CT Scan: It's another type of scan, similar to an X-ray, but more detailed. We don't use it as often for just measuring the prostate, but it can be helpful in some cases.  Camera Test (Cystoscopy): This involves a thin tube with a tiny camera being passed through the urine passage to see inside. It helps us see how the prostate might be pressing into the bladder, though it doesn't directly measure its size.  The best method for you will depend on your symptoms and what we need to find out. We'll discuss it together and decide on the most comfortable and effective way for you. | *EAU patient portal:*  No information available  *AUA patient portal:*  These tests are done to see the size and shape of the prostate. Some BPH scans include:  • Ultrasounds look inside the body and see the size and shape of the prostate.  • Cystoscopy is an exam used to look at the urethra or bladder with a scope.  • Magnetic resonance imaging (MRI) and computed tomography (CT) are more detailed scans. These are done if surgery is necessary to reopen the flow of urine. These scans provide a very clear image of the prostate and surrounding area. It shows exactly how and where the prostate is enlarged. |
|  | 19. What is the classification for prostate enlargement on imaging? | Think of your prostate as a small gland located below your bladder. As men age, this gland can get bigger, and we have ways to look at it using various imaging techniques. Here's how we understand what we see:  Size: Just like we might measure the size of a room, we measure the size of your prostate. A size less than about 30 mL (imagine a small juice box) is usually seen as standard. If it's bigger, it can be slightly, moderately, or significantly enlarged.  Shape & Location of Growth: Your prostate can grow in different patterns. It might grow evenly on both sides, more on one side, or towards the bladder.  Texture: The appearance of your prostate on imaging can be uniform or have different patterns. Sometimes, we might see areas that are a bit harder or softer than the rest.  Other Findings: Occasionally, we might see things like small stone-like structures or fluid-filled pockets in or around the prostate.  Changes in Nearby Areas: If the prostate gets too big, it can press on the bladder. We might notice the bladder wall getting thicker, or there might be more urine left in the bladder after you've gone to the bathroom.  Cancer Check: There's a special system we use, mainly with MRI, to see if there are any signs of cancer in the prostate. It's a scoring system that tells us the likelihood of significant prostate cancer.  It's essential to remember that just because the prostate is big doesn't mean you'll have problems. Some men with a larger prostate have no issues, while others with a smaller one might have symptoms. Our main goal is to understand what's happening and to help you feel better. | No information available in EAU and AUA patient portals |
|  | 20. Can the prostate-specific antigen (PSA) be affected by benign prostatic enlargement? | PSA, or prostate-specific antigen, is a substance made by your prostate gland. We measure its level in the blood to help check on prostate health. Now, while a high PSA can sometimes indicate prostate cancer, there are other reasons it might be elevated.  One common reason is an enlarged prostate, a condition many men experience as they age. This isn't cancer but simply a growth of the gland. When the prostate grows, it can produce more PSA, leading to a higher level in the blood.  Also, if the enlarged prostate becomes a bit inflamed or if you've had certain medical procedures on your prostate recently, this can bump up the PSA level temporarily.  So, if your PSA is high, it doesn't automatically mean you have cancer. It's just one piece of the puzzle. We'll consider other factors, possibly do some more tests, and determine the best course of action together. | *EAU patient portal:*  A protein produced by the prostate which may increase in men with a benign prostatic enlargement, prostatic inflammation, or prostate cancer.  *AUA patient portal:*  Prostate-specific antigen (PSA) blood tests are used to screen for prostate cancer. The PSA blood test checks the level of PSA, a protein made only by the prostate gland. This blood test can be done in a lab, hospital or a provider's office. Avoid sexual activity several days prior to the test, as this may artificially increase the PSA reading. When the prostate is healthy, very little PSA is found in the blood. A rapid rise in PSA may be a sign that something is wrong. A benign (non-cancer) enlargement of the prostate can cause a rise in PSA levels, as can inflammation of the prostate (prostatitis). The most serious cause of a rise in PSA is cancer. |
|  | 21. What is uroflowmetry? | Uroflowmetry is a test we use to see how well you can urinate. Think of it like a speedometer for your urine. It tells us how fast the urine comes out and how much you're able to release.  Here's what you can expect:  Come Ready: We'll ask you to arrive with a full bladder, so you're ready to urinate.  Private Setup: You'll be given privacy to urinate into a special toilet or funnel. This device is connected to a computer that does all the measuring.  The Graph: The computer will make a graph that shows the flow of your urine from start to finish. We look at things like the speed of your flow, how long it takes, and the total amount.  What We Learn: This graph can give us clues about how well your bladder and the tube you urinate through are working. For example, if the flow is slower than usual, it might mean there's some blockage or the muscles are weak.  It's a straightforward and painless test. The main goal is to better understand what's happening when you urinate, so we can help with any issues you might be facing. | *EAU patient portal:*  Uroflowmetry is a simple test that electronically records the rate of urine flow. It is easily done in privacy at the hospital or clinic. This test helps your health care provider check whether the flow of your urine is blocked.  *AUA patient portal:*  Uroflowmetry measures how fast urine flows. This is done to diagnose the problem. It may also be done before surgery. |
|  | 22. What are the normal values of uroflowmetry? | Uroflowmetry is like a speed test for how you urinate. We look at several aspects of how the urine flows, and there are typical values for each, kind of like a 'normal range'. Here's a basic idea of what we usually expect:  Speed of Your Flow (Peak Flow Rate):  For younger men, it's typically around 22-30 mL every second.  For older men, it's around 12-25 mL every second.  For women, it's about 15-25 mL every second.  Average Flow: This is typically about half the speed of the peak flow rate.  Amount of Urine: We usually expect adults to release about a cup to two cups of urine (250-500 mL) each time. But, for the test, we like to see at least a little more than half a cup (around 150 mL) to get a good reading.  Duration of Flow: Most people take between 10-40 seconds to finish urinating.  Time to Reach Peak Flow: Most folks reach their fastest flow within the first 10 seconds of starting to urinate.  Leftover Urine (Post-Void Residual): After urinating, it's ideal to have a very small amount of urine left in the bladder. For most people, especially older adults, this is less than about 1/5 of a cup (50 mL).  Remember, these are just general numbers. Everyone is a bit different, and what's most important is how your results fit into your overall health picture. | *EAU patient portal:*  Normal urine flow is above 15 millilitres per second. If your flow of urine is 10 millilitres per second or less and you experience symptoms, you should see your urologist.  *AUA patient portal:*  No information available |
|  | 23. What is the post-void residual? | Post-void residual, or PVR for short, is a way for us to check how much urine is left in your bladder after you've tried to empty it completely. Think of it like checking how much water remains in a bottle after you've poured most of it out.  We want to measure this because sometimes, for various reasons, the bladder might not empty all the way. Knowing this can help us understand if there's an issue with how your bladder is working.  There are a couple of ways we can check this:  Bladder Ultrasound: This is like taking a picture of your bladder using sound waves. It's a painless process and just involves placing a small device on your lower belly.  Catheterization: This is where we gently insert a thin tube into the bladder to see how much urine comes out. It's more accurate than the ultrasound, but we understand it might be a bit uncomfortable.  If we find that there's a lot of urine left in your bladder after you've tried to empty it, it might mean we need to look into things a bit more to make sure everything's okay. | *EAU patient portal:*  The amount of urine left in the bladder after urination.  *AUA patient portal:*  Post-void residual volume (PVR) measures urine left in the bladder after passing urine. This is done to diagnose the problem. It may also be done before surgery. |
| Conservative management | 24. Are there any behavioral and dietary modifications to manage benign prostatic enlargement? | Managing the symptoms of an enlarged prostate, often called BPE, can be improved with some lifestyle changes. Here are some easy-to-follow suggestions that can help:  Behavioral Tips:  Timed Bathroom Visits: Try going to the bathroom at set times during the day, even if you don’t feel like it. This can train your bladder and reduce sudden urges.  Double-Check: After you finish urinating, wait a moment and try again. This ensures your bladder is completely empty.  Bladder Practice: Gradually increase the time between your bathroom breaks. This can help your bladder hold more over time.  Evening Drinks: If getting up at night to pee bothers you, try drinking less in the evening.  Pelvic Exercises: There are exercises, called Kegels, that can strengthen the muscles around the bladder. They can help with bladder control.  Keep Moving: Regular walks or exercises can help with bladder health and reduce the chance of constipation, which can make prostate symptoms worse.  Handle Constipation: Make sure your diet has enough fiber. A full bowel can put pressure on your bladder.  Stay Warm: Cold can make you pee more often. So, bundle up when it's chilly!  Dietary Tips:  Watch Caffeine and Alcohol: These can make you pee more often and can irritate your bladder. Consider reducing your intake.  Eat Well: Focus on fresh fruits, vegetables, and grains. Some foods, like tomatoes and nuts, might be particularly good for prostate health.  Stay Hydrated: Drink water throughout the day, but try not to drink too much at one time.  Spicy and Acidic Foods: If you notice foods like these irritate your bladder, it might be good to eat them less often.  Supplements: There are supplements like beta-sitosterol that some people find helpful. But always talk to your doctor before starting any new supplement.  Cut Down on Salt: Too much salt can make you pee more. Watch out for processed foods which tend to be high in salt.  Remember, these are just guidelines. Everyone is different, so you might find some tips more helpful than others. And always let your doctor know about any significant changes you're making. The aim is to make daily life more comfortable for you! | *EAU patient portal:*  Drink at least 1 litre every day and discuss with your doctor if you can drink more  Drink more if you live in a hot climate or do a lot of physical exercises  Drink less before and during long trips  Drink less in the evening to avoid getting up at night to urinate  Avoid alcohol and caffeine because they increase urine production and irritate the bladder  Try to exercise 2 or 3 times a week. Lack of movement can make it more difficult to urinate and cause urinary retention  Have a balanced and varied diet  Always try to keep your lower abdomen dry and warm. If you go swimming, bring an extra set of dry clothes and change as soon as you are out of the water. Dampness and cold may increase the need to urinate and can cause a urinary infection  Sometimes urine sprays and it can wet the toilet seat or the bathroom floor. Some men prefer to sit down when urinating to avoid this, while others prefer to urinate in a cup and empty it in the toilet  Apart from following general lifestyle advice, you can actively manage the symptoms caused by BPE in your everyday life. Self-management can reduce symptoms and keep your condition stable.  Completely empty your bladder each time you urinate. It may help if you sit down  If you feel your bladder is not empty after urinating, try again after 5 minutes  Use a small pad to catch involuntary urine loss  After urinating, press under the scrotum with your fingers onto the urethra and then slide your fingers from the base to the tip of the penis to squeeze out the last drops of urine. This will help to avoid wetting your underwear  Use breathing exercises to distract yourself from the feeling of urgency  Apply pressure to your penis or perineum (Fig. 1) to divert your attention from urination  Encourage yourself to “hold on” longer when you feel the urgency to urinate. This will train your bladder to keep more urine so that you will urinate less often  Avoid constipation by adapting your diet  Avoid sudden exposure to cold weather and always try to keep your lower abdomen warm  Keep a bladder diary  *AUA patient portal:*  Diet and exercise are often recommended as a way to prevent or manage symptoms. |
|  | 25. What is watchful waiting in benign prostatic enlargement? | Watchful waiting is like taking a 'wait and see' approach with certain medical conditions. Instead of jumping into treatment right away, we keep a close eye on things. Here's what you should know:  Keeping an Eye on Things: Instead of starting treatment immediately, we'll regularly check on how you're doing and if the condition changes.  Routine Check-ins: You'll have regular appointments, and sometimes we'll do tests to make sure things are stable.  Why We Choose This: Sometimes, the potential side effects of a treatment might be more risky than the condition itself, especially if it's not causing you major problems or if it's progressing very slowly.  Switching Gears: If we notice that things are getting worse or if you start feeling differently, we can then decide to begin treatment.  Staying Informed: We'll talk through everything, so you understand the plan. And always let us know if you notice any changes in how you feel.  This approach is often used for conditions that grow slowly and might not cause any problems for a long time, like certain types of cancer or mild prostate enlargement. | *EAU patient portal:*  You may wish to consider ‘watchful waiting’ if your symptoms are mild to moderate and they aren’t bothersome enough to noticeably affect your quality of life.  A watchful waiting programme involves:  Assessment of your symptoms during a consultation appointment with a doctor  A physical examination  Blood and urine tests  Education and advice about your condition  Ongoing support with managing your BPE  During watchful waiting, mild to moderate BPE symptoms can often be treated with lifestyle changes such as:  Drinking at least 1 litre of fluid every day.  Drinking less fluid before going to bed or before long trips to reduce the need to urinate during these times.  Drinking less alcohol, caffeine, fizzy drinks and artificial sweeteners, which can irritate the bladder and make your symptoms worse.  Taking regular exercise (at least 2-3 times a week). Lack of movement can make it more difficult to urinate.  Having a balanced and varied diet.  Keeping your lower abdomen warm and dry. Cold and damp increases the urge to urinate.  If your urine sprays when you go to the toilet, it can be helpful to sit down to urinate instead of standing or if you prefer to stand, to urinate into a cup and pour that into the toilet.  Discuss with your doctor, all medication you are taking for any condition as some medicines, such as ‘water tablets’ (called diuretics), may cause urinary symptoms to worsen.  You will be offered regular check-ups as part of watchful waiting, to make sure that your condition has not got any worse, or more troublesome to you. If it has, your doctor can discuss treatment options that are available to you.  *AUA patient portal:*  Often, BPH may only require active surveillance. This means your BPH will be closely watched but not actively treated and may be monitored with regular visits to your urologist. A yearly exam is common. Your health care provider will look for worse or new issues before suggesting anything else. Active surveillance is best for men with mild to moderate symptoms. It is also an option for men who are not bothered by the effects of BPH. |
| Pharmacotherapy | 26. What are the indications of oral therapy in a patient with benign prostatic enlargement? | Benign Prostatic Enlargement (BPE) is when the prostate gland grows larger than usual. This growth can sometimes cause issues with urination. There are pills available that can help with this. Let's talk about when we might consider these medications:  Symptoms Are Noticeable: If your prostate-related symptoms are moderate to severe, making daily tasks uncomfortable, we might think about medication.  It's Affecting Your Daily Life: If you feel your quality of life is reduced because of frequent bathroom visits or difficulty urinating, medication could be beneficial.  Lifestyle Changes Didn't Help: Sometimes, dietary changes or exercises can help, but if they don't, we can consider medicine.  Related Health Issues: If you're having problems like repeated urinary infections, inability to pass urine suddenly, bladder stones, or issues with your kidneys because of BPE, medicine might be necessary.  Size of Your Prostate: Some medicines work best if the prostate is larger, while others work regardless of its size.  Chance of It Getting Worse: If tests show that your prostate may continue to grow or cause more problems, we might start medication sooner.  Your Preference: After understanding the benefits and possible side effects, you might decide to start medication as your first choice of treatment.  Now, the kinds of pills we usually consider are:  Alpha-blockers: These help relax the muscles around your prostate, making it easier to pee. Common ones include tamsulosin and alfuzosin.  5-alpha reductase inhibitors: Examples are finasteride and dutasteride. They help shrink the prostate size by affecting certain hormones.  PDE5 inhibitors: Tadalafil is an example. You might know them as treatments for erection issues, but they can also help with prostate symptoms.  Combination: Sometimes, we might give you two different pills because together they work better, especially if your prostate is quite large and causing more severe symptoms.  Remember, everyone is different. We'll choose a treatment based on what's best for you, considering your symptoms, the size of your prostate, and other health factors. | *EAU patient portal:*  You have been diagnosed with benign prostatic enlargement (BPE) and your doctor recommends drug treatment. This treatment is advised when the symptoms are bothersome and affect your quality of life. This section describes different drug treatments, which you should discuss with your doctor. Together you can decide which approach is best for you.  *AUA patient portal:*  No information available |
|  | 27. What are alpha 1-adrenoceptor antagonists (α1-blockers)? | Alpha 1-Adrenoceptor antagonists, often called α1-blockers, are medications that help in two main ways:  Blood Pressure Control: They can help widen your blood vessels, making it easier for blood to flow and potentially lowering your blood pressure.  Easing Urination Problems: If you have an enlarged prostate, which is a common condition in older men, these drugs can relax the muscles around the bladder and prostate. This helps improve urine flow and reduce symptoms like the frequent need to pee, especially at night, or feeling like you can't empty your bladder completely.  Here are some common names you might hear:  Tamsulosin (like Flomax)  Alfuzosin (like Uroxatral)  Terazosin (like Hytrin)  Doxazosin (like Cardura)  Silodosin (like Rapaflo)  Keep in mind that while these medications can be very helpful, they can sometimes cause side effects like dizziness or feeling light-headed, especially when standing up quickly. It's essential to talk with your doctor about any concerns or if you notice any changes while on these medications. | *EAU patient portal:*  For some men experiencing nocturia, their doctor may prescribe medicines called alpha blockers. Alpha blockers relax the muscle in the prostate gland or reduce the size of the prostate. This is because the prostate is close to the bladder and these medicines can aid the process of fully emptying the bladder. Alpha blockers are not specifically designed to treat nocturia. While taking these medicines may improve bladder emptying during the day, there is limited evidence to suggest that they can reduce the need to urinate at night. Your doctor can provide more information on whether these medicines are right for you.  Side effects of alpha blockers are mild, and most men do not experience any. In those who do, loss of strength, dizziness, and slightly reduced blood pressure, are possible but temporary. It is possible that these medicines can affect sexual function (sex drive and ejaculation).  *AUA patient portal:*  Alpha blockers are pills used to relax the muscles of the prostate and bladder to reduce BPH symptoms. They do not shrink the prostate but they may improve urine flow if there is a blockage. One benefit of alpha blockers is they start to work right away. |
|  | 28. What is the efficacy of alpha 1-adrenoceptor antagonists (α1-blockers) for benign prostatic enlargement treatment? | Alpha 1-Adrenoceptor antagonists, or α1-blockers for short, are medications often prescribed to men with an enlarged prostate, a condition we sometimes call BPE or BPH. Here's what you should know about how they work:  Quick Symptom Relief: These medications can help improve common urinary symptoms like trouble starting to pee, a weak stream, or feeling like you still have to go after you've finished. Many men notice improvements within a few days to weeks.  Boosting Urine Flow: Not only can they relieve symptoms, but these medications also help increase the flow of urine, making it easier to empty your bladder.  Side Effects: While many men tolerate these drugs well, some might feel dizzy or lightheaded, especially when getting up from a seated or lying position. It's always good to be aware and move slowly.  Comparison with Other Medications: While α1-blockers work quickly, there are other prostate medications that act slower but can shrink the prostate over time. Sometimes, doctors prescribe both types together for the best results.  Long-Term Use: These medications help with symptoms, but they don't stop the prostate from growing or cure the condition. Some men might need additional treatments or adjustments to their medication over time.  Different Types: There are a few different α1-blockers available, and while they all help with prostate symptoms, some might be a better fit for you than others. If one doesn't seem to work well or if you experience side effects, let your doctor know. They might switch you to another one.  Remember, it's crucial to stay in touch with your doctor and let them know how you're feeling on the medication. Regular check-ups will help ensure that you're getting the best treatment for your condition. | No information available in EAU and AUA patient portals |
|  | 29. What are the most common side effects of alpha 1-adrenoceptor antagonists (α1-blockers)? | Alpha 1-Adrenoceptor antagonists, or α1-blockers, are generally well-tolerated, but like all medications, they can have side effects. Some of the most common side effects include:  Orthostatic Hypotension: This is a sudden drop in blood pressure when a person stands up from a sitting or lying position. It can lead to dizziness or feeling lightheaded, and in some cases, fainting.  Dizziness: Apart from orthostatic hypotension, some people might just feel generally dizzy when taking α1-blockers.  Fatigue: Some people might feel unusually tired or weak.  Headache: Some patients report headaches when starting on these medications.  Nasal Congestion: The medications can cause the blood vessels in the nasal passages to expand, leading to stuffiness or congestion.  Retrograde Ejaculation: This is when semen goes backward into the bladder during ejaculation instead of out through the penis. It's harmless and doesn't affect orgasm, but it can reduce the volume of semen expelled and might interfere with fertility.  Palpitations: Some people might feel like their heart is beating too hard, too fast, skipping a beat, or fluttering.  Swelling in the Ankles or Feet: This is due to fluid buildup, also known as peripheral edema.  Gastrointestinal Symptoms: Some patients might experience nausea, diarrhea, or abdominal discomfort.  It's worth noting that while these side effects are possible, they don't occur in everyone, and some people might experience them more mildly than others. It's always essential for patients to discuss any side effects or concerns with their healthcare provider. Adjustments in dosage or switching to another medication might be necessary in some cases. | *EAU patient portal:*  Loss of strength, dizziness, and slightly reduced blood pressure, are possible but temporary. It is possible that these medicines can affect sexual function (sex drive and ejaculation).  *AUA patient portal:*  Side effects may include dizziness, lightheadedness, fatigue and difficulty ejaculating. Men with moderate to severe BPH and men who are bothered by their symptoms are good candidates. Alpha blockers are not a good choice if you plan to have cataract surgery soon. |
|  | 30. What are 5α-reductase inhibitors? | 5α-reductase inhibitors are medications that help manage certain conditions by acting on male hormones. Here's a breakdown:  What They Do: These medicines reduce the action of an enzyme called 5α-reductase. This enzyme turns the male hormone testosterone into a stronger form called DHT. DHT is involved in making the prostate gland grow and in hair loss on the scalp.  Why We Use Them:  Prostate Issues: One of the main uses is to treat an enlarged prostate, a condition where the prostate gland grows bigger and can cause problems with urination.  Hair Loss: These drugs can also help treat hair loss in men, which is caused by the same DHT hormone affecting hair follicles.  Common Medications:  Finasteride: Used for both an enlarged prostate and hair loss.  Dutasteride: Mainly used for an enlarged prostate.  Things to Keep in Mind:  Like all medicines, there can be side effects. Some men might notice a decrease in sexual desire, difficulty in getting or keeping an erection, or a decrease in the amount of semen.  If you're planning to have a family or if there's a chance of pregnancy, it's important to note that these medications can affect male babies during pregnancy. So, women who are pregnant or might become pregnant should avoid handling these tablets.  Remember, it's always a good idea to talk to your doctor about any concerns. They can guide you on whether this medication is right for you and discuss any potential side effects. | *EAU patient portal:*  Group of drugs which prevent the prostate from growing and may even shrink it. These drugs work better in prostates larger than 40 mL and are only prescribed when prostate enlargement symptoms cause significant problems.  *AUA patient portal:*  Pills that can increase urine flow and shrink the prostate by blocking DHT. DHT is a male hormone that can build up in the prostate and may cause prostate growth. These drugs may lower the risk of BPH problems and the need for surgery. |
|  | 31. What is the efficacy of 5α-reductase inhibitors for benign prostatic enlargement treatment? | 5α-reductase inhibitors are medications that work on the male hormones to treat an enlarged prostate. Here's how they help:  Shrink the Prostate: Think of your prostate as a balloon that's getting too big. These medicines help let some air out, making it smaller. This can help you urinate more comfortably.  Easier Urination: As the prostate gets smaller, many men find it easier to pee and don't need to go as often, especially during the night.  Avoiding Other Problems: By keeping the prostate in check, these medications can prevent some severe issues. For instance, they lower the chances of suddenly not being able to pee at all or needing surgery for the prostate.  Working with Other Medications: Sometimes, doctors recommend taking another medicine alongside this one. This combo can give better relief from pee problems.  Patience is Key: While you might start feeling better in a few months, the best results, like a much smaller prostate, can take a bit longer - maybe 6 months or more.  Long-term Benefits: If you keep taking the medicine as your doctor recommends, it will continue to help over time, keeping urinary troubles at bay.  Remember, while these medicines are useful, they're not for everyone. Your doctor will consider your specific situation, how bad your symptoms are, and other factors before recommending them. | No information available in EAU and AUA patient portals |
|  | 32. What are the most common side effects of 5α-reductase inhibitors? | 5α-reductase inhibitors are medicines that help treat an enlarged prostate. Like all medicines, they can have side effects. Here's what some men have noticed:  Changes in Sexual Feelings or Actions:  Desire: Some men feel less interested in sex.  Performance: Some find it harder to get or keep an erection.  Ejaculation: Some notice less fluid when they ejaculate.  Breast Changes: A few men have felt their breasts grow or become tender.  Hair Growth: Oddly enough, while these medicines can help with prostate issues, they can also make hair grow. This is why they're sometimes used for baldness!  Allergic Signs: It's rare, but some men might get a rash, itchiness, or swelling, especially around the lips and face.  Mood Changes: A very small number of men have felt more down or depressed.  PSA Test: This is a blood test to check on the health of your prostate. This medicine can make the PSA number go down. So, if you're having this test, let your doctor know you're on this medicine, as it can affect the results.  Remember, not everyone will feel these side effects. If you notice any or if they bother you, it's essential to talk to your doctor. They can help manage them or discuss other treatment options. | *EAU patient portal:*  The side effects of these drugs are mainly related to sexual function. They may include reduced sexual drive, erectile dysfunction, and problems with ejaculation. However, incidence of sexual dysfunction is low in these patients and even decreased with treatment duration. About 1-2% of men experience breast enlargement or nipple tenderness. Side effects are not very common and disappear when the treatment is stopped.  *AUA patient portal:*  Side effects include erectile dysfunction and reduced libido (sex drive). You must keep taking the pills to prevent symptoms from coming back. These may be best for men with very large prostate glands.  These prescription drugs may take many months to become fully effective and include dutasteride and finasteride. |
|  | 33. What are phosphodiesterase 5 inhibitors? | Phosphodiesterase type 5 inhibitors, or PDE5 inhibitors for short, are medicines mainly used to help men with erection problems. They work by improving blood flow to the penis. Here's a quick breakdown:  Sildenafil (Viagra): This medicine helps men get and keep an erection. It was actually first made to help with heart issues!  Tadalafil (Cialis): Apart from helping with erections, it can also help with prostate problems and a lung condition called pulmonary arterial hypertension. One pill can work for up to 36 hours, so some people call it 'the weekend pill.'  Vardenafil (Levitra, Staxyn): Another medicine to help with erections.  Avanafil (Stendra): A newer drug for erection problems. It can start working faster than some of the others.  In addition to helping with erection problems, some of these medicines, like Cialis, can help with symptoms men might feel when they have an enlarged prostate, like going to the bathroom too often.  Like all medicines, these can have side effects. Some common ones are headaches, feeling warm and red in the face, stuffy nose, or an upset stomach. Always chat with your doctor before starting these, to make sure they're right for you. | *EAU patient portal:*  Group of drugs used for the treatment of erectile dysfunction. These drugs can also improve some symptoms caused by BPE.  *AUA patient portal:*  No information available |
|  | 34. What is the efficacy of the phosphodiesterase 5 inhibitors for benign prostatic enlargement treatment? | You know those medicines like Cialis that help with erection problems? It turns out they can also help with some prostate issues. Here's a rundown:  Symptom Relief: These medicines can help with the annoying symptoms you might have from an enlarged prostate. This includes needing to go to the bathroom often, especially at night, or feeling a strong urge to pee.  How It Works: We're not entirely sure of all the details, but it seems these drugs help relax certain muscles and might even reduce swelling in the prostate.  Two Birds, One Stone: If you have both erection problems and bathroom troubles from your prostate, one of these pills (like Cialis) could potentially help with both.  Side Effects: Generally, these medicines are safe, but some men might get headaches, some flushing in the face, or a bit of back pain. Also, if you're on heart medicines, especially nitrates, it's essential to talk to your doctor as there could be interactions.  Compared to Other Prostate Meds: There are other medicines for prostate symptoms too, like Flomax. Both can help, but they work in different ways. If you also have erection issues, then a pill like Cialis might make sense.  In short, if you're having trouble with going to the bathroom often or with erections, this kind of medicine could help. But it's important to chat with your doctor to figure out what's best for you. | No information available in EAU and AUA patient portals |
|  | 35. What are the most common side effects of phosphodiesterase 5 inhibitors? | Those medications that can help with erection problems, like Viagra or Cialis, are generally safe for most people. But, like all medicines, they can have some side effects. Here's what you might notice:  Headache: This is the most common one.  Feeling Warm and Red: Especially on your face or chest.  Upset Stomach: Like heartburn or stomach discomfort.  Stuffy Nose: Just like when you have a cold.  Back Pain or Muscle Aches: Some people feel a bit sore.  Changes in Vision: Rarely, things might look blueish or it might be hard to tell some colors apart. If you have sudden vision problems, seek help right away.  Hearing Issues: Very rarely, there could be a sudden decrease in hearing. Again, get medical help if this happens.  Dizziness: You might feel lightheaded, especially when standing up.  Long-lasting Erection: If it lasts more than 4 hours, head to the hospital. It sounds funny, but it can actually be harmful.  And remember, always let your doctor know what other medicines you're on. Some can interact with these pills, especially certain heart meds.  If you ever feel something isn't right after taking the medication, it's always a good idea to chat with your doctor. | *EAU patient portal:*  Side effects such as headache, back pain, dizziness, and indigestion. PDE5Is should not be used in combination with several drugs, including the alpha-blockers doxazosin or terazosin. They should also not be used in men who have specific heart problems. Men who have problems with their blood pressure or who have kidney failure should also not take PDE5Is. If you experience loss of vision when taking PDE5Is, you should go to your doctor.  *AUA patient portal:*  No information available |
|  | 36. What are plant extracts/phytotherapy for benign prostatic enlargement treatment? | Plant extracts or herbal remedies are often used by people to help with an enlarged prostate. Here are some of the popular ones:  Saw Palmetto: This is a small palm tree and its fruit extract is commonly used for prostate problems. Some people find it helpful, but the studies are mixed on how effective it really is.  Pygeum: This comes from the bark of an African tree. It's been traditionally used in some parts of the world to help with urination issues.  Beta-sitosterol: These are substances found in many plants. Some people believe they help with urination flow.  Stinging Nettle: Don't worry, it won't sting you! The roots of this plant have been used for urinary symptoms.  Pumpkin Seed: Some believe that eating these seeds or taking supplements can help reduce prostate size and improve symptoms.  Rye Pollen Extract: This one might help reduce the number of times you need to go to the bathroom during the night.  While some folks swear by these remedies, it's important to remember that their effectiveness isn't proven like traditional medicines. Always chat with your doctor before trying any new supplement, as they might interact with other meds you're taking or have unexpected side effects. | *EAU patient portal:*  Herbal drugs are made of plant extracts. Many types of roots, seeds, pollen, bark, or fruits can be used alone or in combination to make these drugs. The most widely used extracts in herbal drugs are taken from:  Pumpkin seeds (Cucurbita pepo)  South African star grass (Hypoxis rooperi)  Bark of the African plum tree (Pygeum africanum)  Rye pollen (Secale cereal)  Berries of the American dwarf palm (Serenoa repens)  Roots of the stinging nettle (Urtica dioica)  It is not entirely clear how these herbal drugs work to relieve symptoms caused by BPE. It is also not clear how efficient they are. The quality of herbal drugs can vary greatly. Because there are so many herbal drugs available, no specific recommendation about their use can be made. Inform your doctor during consultation if you take any herbal drugs to relieve your symptoms.  *AUA patient portal:*  Phytotherapies or herbal therapies are not recommended by health care providers. One popular herb is saw palmetto. Several important studies show they do not work. Also, the quality and purity of supplements vary. |
|  | 37. What is the efficacy of the plant extracts/phytotherapy for benign prostatic enlargement treatment? | Many people are interested in using natural or herbal treatments for prostate enlargement. Here's a quick breakdown of what the research says about some popular options:  Saw Palmetto: This is a widely-used herbal remedy. Some studies say it helps, while others don't see much difference. It's a bit like a seesaw, with opinions going back and forth.  Pygeum: Made from the bark of an African tree, some people find it helpful, but more studies are needed to confirm its benefits.  Beta-sitosterol: Found in many plants, some studies suggest it might help with urine flow. But again, results vary.  Stinging Nettle: Used traditionally in some parts of the world, it might work better when combined with other herbs. By itself, there's limited evidence.  Pumpkin Seed: While some believe it's beneficial, especially when paired with other treatments, the evidence is still limited.  Rye Pollen Extract: This one might help reduce nighttime bathroom trips for some, but again, we need more research.  The bottom line is that while many folks believe in these herbal remedies, the science isn't always clear-cut. If you're thinking about trying any of them, it's essential to chat with your doctor first. Even natural treatments can have side effects or interfere with other medicines you're taking. | No information available in EAU and AUA patient portals |
|  | 38. What are the most common side effects of plant extracts/phytotherapy for benign prostatic enlargement treatment? | Some people turn to natural or herbal treatments for prostate issues, believing they might be gentler than regular medicines. While they can be milder, it's essential to know they can also have side effects. Here's a quick summary:  Saw Palmetto: Some folks experience stomach issues like nausea or diarrhea. A few might feel dizzy, tired, or get a headache. On rare occasions, it might affect the liver or pancreas.  Pygeum: This can occasionally cause stomach upset, diarrhea, or sometimes nausea.  Beta-sitosterol: This might cause some stomach problems, gas, or even affect sexual drive in rare cases.  Stinging Nettle: Mostly, it's just mild stomach issues. If you touch the fresh plant, it can sting, but the supplement you take doesn't have this effect.  Pumpkin Seed: Very few side effects, but a tiny number of people might get an upset stomach.  Rye Pollen Extract: Most people are fine with this, but a few might get some stomach discomfort.  Remember, just because something is natural doesn't mean it's free from side effects. Everyone's body reacts differently. If you're thinking about trying any of these, chat with your doctor first to make sure it won't interfere with other things you're taking or any other health conditions you might have. | *EAU patient portal:*  Side effects of herbal drugs are mild and many men do not experience any side effects at all. Gastro-intestinal discomfort (for instance bloating or constipation) is the most common complaint.  *AUA patient portal:*  No information available |
|  | 39. What are the indications for combining oral therapies to treat benign prostatic enlargement? | Sometimes, one medication might not be enough to help with the symptoms of an enlarged prostate. In such cases, we consider combining different medicines. Here are the reasons why we might do that:  Not Enough Relief with One Medication: If you've been taking one medication and still have symptoms, we might add another one to give you more relief.  Higher Risk of Bigger Problems: If tests show that your prostate is quite large or if a blood test called PSA is high, combining medicines can help reduce the risk of more severe issues like suddenly not being able to pee at all or needing surgery in the future.  Both Prostate and Erection Problems: Some men have both an enlarged prostate and trouble with erections. There's a combination of medicines that can help with both at the same time.  Reducing Side Effects: By using two medicines, sometimes we can use lower doses of each, which might mean fewer side effects for you.  After a Blockage: If you've had a sudden situation where you couldn't pee at all while on one medication, we might add another to prevent it from happening again.  Different Types of Symptoms: If you find yourself running to the bathroom often and also having trouble starting the urine flow, combining medications can help with both types of problems.  Remember, combining medications can be beneficial, but it also means there's a chance for more side effects. It's crucial to talk with your doctor about how you're feeling, and if anything changes, let them know right away. | *EAU patient portal:*  The combination of alpha-blockers with 5ARIs is recommended if:  your prostate is larger than 40 mL;  your prostate-specific antigen level is 1.5 ng/mL or higher;  your symptoms are severe, and you have slow flow of urine.  This combination of drugs is only advised for long-term treatment.  The combination of alpha-blockers and MRAs or beta-3 agonists is recommended if you have symptoms such as an increased or sudden need to urinate and your symptoms have not improved when taking one type of drug.  *AUA patient portal:*  No information available |
|  | 40. What is the recurrence of benign prostatic enlargement after oral medications? | Certainly! When discussing the likelihood of symptoms returning after taking oral medications for Benign Prostatic Enlargement (BPE), here's a simplified explanation you might find helpful:  Different Medications Work Differently: There are various kinds of pills for BPE. Some help by relaxing the muscles in your prostate and bladder to ease symptoms, while others work to make the prostate smaller. How well these medicines work and how long they keep your symptoms at bay can vary.  Your BPE Condition Matters: If your prostate enlargement is more advanced, there's a chance that the symptoms might come back more easily, even with medication.  Everyone Responds Uniquely: People's bodies react differently to medications. Some might find great relief and fewer symptoms coming back, while others might not get as much benefit from the same medication.  Taking Your Medicine Regularly Is Key: It's really important to take your medicine exactly as prescribed. If you miss doses or don’t take it regularly, your symptoms are more likely to return.  Medication Effectiveness Over Time: Over time, some medications might not work as well as they did at first. This could mean your symptoms might reappear.  Remember, these medications manage the symptoms but don't cure the underlying condition. It’s essential to keep in touch with your doctor, let them know how you're doing, and discuss any changes in your symptoms. They can adjust your treatment as needed to help you manage your condition better. | *EAU patient portal*:  BPE cannot be completely cured and all medical and surgical treatments that are available focus on reducing the symptoms as far as possible. BPE symptoms and how bothersome they are can change over time. You may or may not decide to have further or more invasive treatment depending on your circumstances or how much the symptoms affect your daily life.  Your doctor will help you to make informed decisions about how you decide to treat your BPE and advise you of any new treatment options available so that your BPE is as well-managed as it can be, and you are able to enjoy your relationships and participate in all aspects of your life.  *AUA patient portal*:  No information available |
| Surgical management | 41. When does a patient need surgery for benign prostatic enlargement? | Think of your prostate as a donut that's gotten too big, squeezing the tube (urethra) running through it, making it harder for urine to flow. Sometimes, medicines can help, but in certain situations, we might need to consider surgery to open up that tube and improve the flow. Here's when we typically think about surgery:  Medications Not Helping Enough: If you've tried medications and they aren't giving you enough relief, surgery might be the next step.  Suddenly Can't Pee: If there's ever a time when you just can't pee at all, that's a big sign we might need to consider surgery.  Stones in the Bladder: Imagine tiny rocks forming because the bladder isn't emptying all the way. If you have these, we might need to remove them and also think about surgery for the prostate.  Frequent Bladder Infections: If you keep getting bladder infections because the bladder isn't emptying well, surgery might help.  Bladder Damage: If the bladder has to push really hard for a long time to get the urine out, it can get tired and damaged. Surgery can help prevent further damage.  Blood in the Pee: If you notice blood in your urine and it's because of the prostate squeezing too hard, surgery might be needed.  Really Big Prostate: If your prostate is super-sized, medicines might not help as much, and surgery could be a better option.  Your Choice: Sometimes, you might just prefer to have surgery rather than taking pills every day or if you don't like how the medicines make you feel.  Remember, surgery is just one option. It's essential to chat with your doctor about what's best for you, understanding the pros and cons. | *EAU patient portal*:  Most men with urinary symptoms arising from BPE do not need to have surgery, but it may be an option if other treatments have not worked, or if your symptoms have worsened and you feel they are noticeably affecting your quality of life. Your doctor will discuss whether surgery is appropriate for you, and the potential options so that you can make an informed decision. When your symptoms get worse, even if you already receive drug treatment  When you have complications of BPE or if you are at risk of getting them. Complications include:  Kidney failure  Dilatation of your kidneys  Inability to urinate (urinary retention)  Recurring urinary tract infection  Recurring blood in the urine  Bladder stone  If you do not tolerate drug treatment very well  If you prefer surgery over drug treatment  *AUA patient portal*:  In severe cases of BPH or when other options fail, more invasive surgery is recommended . Surgery is best if you:  • Are unable to pass urine  • Have kidney damage  • Have frequent urinary tract infections • Have a lot of bleeding  • Have stones in the bladder |
|  | 42. Which surgical procedures are available for benign prostatic enlargement? | Alright, if you're considering surgery for your enlarged prostate, there are several options available. Think of your prostate like a donut that's grown too big and is squeezing the tube (urethra) in the middle. We have different ways to relieve that squeeze:  TURP (Scooping Out Method): We insert a tiny tool into the pee tube and scoop out the extra prostate tissue. It's like hollowing out the inside of the donut. This is one of the most common methods.  TUIP (Cutting Method): Instead of removing tissue, we make a couple of small cuts in the prostate to open it up and ease the squeeze.  Open Prostatectomy (Open Surgery): We make a cut on your lower belly and remove a big chunk of the prostate directly. This is mainly for really large prostates.  Laser Treatments (like HoLEP or GreenLight): We use a laser to either cut out or melt away the extra prostate tissue.  UroLift (Clipping Method): We put tiny clips in to hold the squeezed part of the prostate away from the pee tube.  Rezūm (Steam Method): We inject steam into the prostate, which causes the extra tissue to shrink over time.  Aquablation (Water Jet Method): We use a high-speed stream of water to wash away the extra prostate tissue.  Microwave or Radio Waves (Heat Methods): We use heat, either from microwaves or radio waves, to shrink the prostate tissue.  The best method for you will depend on how big your prostate is, your overall health, and what your doctor recommends. Each method has its benefits and potential risks, so it's essential to have a good discussion with your doctor about what's right for you. | *EAU patient portal*:  The main procedures are:  Transurethral resection of the prostate (TURP)  Transurethral incision of the prostate (TUIP)  Open prostatectomy  Laser vaporisation of the prostate  Laser enucleation of the prostate  Prostatic urethral lift  *AUA patient portal*:  The main procedures are:  Transurethral resection of the prostate (TURP)  Transurethral incision of the prostate (TUIP)  Open prostatectomy  Laser vaporisation of the prostate  Laser enucleation of the prostate (HoLep, Thulium)  Prostatic urethral lift |
|  | 43. What are the general complications after prostate surgery? | After having prostate surgery, it's important to know that, like any surgery, there are potential side effects and complications. I'll go over the common ones:  Bleeding: Sometimes, there can be more bleeding than expected. Rarely, it might be necessary to get some extra blood or even have another surgery.  Infection: Any surgery carries a risk of infection. If you notice increased pain, swelling, redness, or pus, it might be an infection, and we should address it.  Difficulty Holding Urine: Some men might find it challenging to control their urine for a short time after surgery. This usually gets better.  Trouble Emptying the Bladder: Even after surgery, some might still have difficulty emptying the bladder completely.  Changes in Sexual Function: The surgery might affect erections or the way ejaculation feels. Instead of going out, semen might go backward into the bladder – this doesn't hurt but can be surprising.  Narrowing of the Urethra: Sometimes, scar tissue can form and make the tube you pee through (urethra) narrower.  TUR Syndrome: This is specific to a type of surgery called TURP. It's rare but can make you feel sick if the body absorbs too much of a certain fluid used during the procedure.  Injury to Nearby Parts: There's a small chance of injury to the area near the prostate.  Blood Clots: Being less active after surgery and the surgery itself can increase the risk of clots forming in the legs. We take steps to minimize this risk.  Allergic Reaction: Rarely, there might be a reaction to the medications used during the surgery.  Remember, these complications don't happen to everyone. Our team is here to ensure everything goes smoothly and address any issues promptly. Always let us know if you feel something isn't right after your surgery. | *EAU patient portal*:  The most common temporary side-effects of surgery for BPE include:  Infections  Risk of bleeding  Being unable to pass urine  Scarring that narrows the urethra  Temporary pain when urinating  If you experience any of the above side-effects, they should resolve a short time after surgery but please speak to your doctor if you are concerned, or if the effects seem to be lasting for longer than a few days post-surgery.  Permanent retrograde ejaculation (where semen enters the bladder instead of emerging through the penis) is a possible long-term to permanent side-effect of surgery and this should be discussed with your doctor.  *AUA patient portal*:  Your orgasm is not expected to change. In some cases, men can experience an issue where semen enters the bladder rather than out the penis (retrograde ejaculation).  Infection, bleeding and incontinence may also occur after some BPH treatments. In some cases, scar tissue may form.  Side effects vary with the type of treatment you choose and most side effects are temporary. Some men need further or new treatment for their BPH symptoms after initial treatment. |
|  | 44. What is a transurethral resection of the prostate? | TURP stands for "transurethral resection of the prostate." It's a surgery mainly used to help men who have trouble peeing because their prostate gland has grown larger. This growth isn't due to cancer; it's a common condition many men experience as they age, called benign prostatic hyperplasia or BPH.  Why might you need it?  If your prostate has grown larger, it might be pressing on the tube you pee through (urethra). This can cause:  Trouble starting to pee  A weak stream or dribbling afterward  Feeling like you always need to pee  How does TURP work?  During the procedure:  You'll be given medicine so you won't feel any pain. This could be anesthesia that puts you to sleep or numbs just the lower part of your body.  The doctor uses a special instrument that goes into the penis without making any cuts on the outside. This instrument has a tiny camera and a tool to remove the extra prostate tissue.  The doctor trims away the inner part of the prostate to make more room for urine to flow easily.  After the surgery, a soft tube might be placed in the penis for a short time to help you pee while healing.  What are the benefits?  After recovery:  Most men find it much easier to pee.  It often provides long-term relief.  Are there any risks?  Like all surgeries, there are some risks. These might include:  Some bleeding  Chance of an infection  Rarely, problems with getting an erection or changes in ejaculation  What happens after the surgery?  You might stay in the hospital for a day or two. It's common to see blood in the urine at first, but this should get better. You'll need to take it easy and might have a follow-up appointment to see how you're healing.  Remember, every person is different. It's important to talk to your doctor about any concerns, ask questions, and understand the potential benefits and risks before deciding on the procedure. | *EAU patient portal*:  This is the most common surgery for BPE and is usually done under a general anaesthetic or spinal anaesthetic so you will not feel any pain during the procedure.  During the procedure, a thin device called a resectoscope is inserted through the urethra. The resectoscope has a wire loop at the end of it which uses a high-frequency electrical current to cut the prostate tissue. It also has a camera which allows the surgeon to see a high-quality image of the prostate on a video monitor. The surgery involves removing the section of prostate tissue that is blocking the flow of urine.  At the end of the operation, a catheter is placed in your bladder to help drain urine while you recover from the surgery.  *AUA patient portal*:  Transurethral resection of the prostate (TURP) is a common surgery for BPH. TURP uses electric current or laser light to cut and remove tissue. This is done with anesthesia and a tool called a resectoscope inserted through the penis. The resectoscope provides light, irrigating fluid and an electrical loop. The loop cuts tissue and seals blood vessels. The removed tissue is flushed into the bladder and out of the body with a catheter |
|  | 45. What are the complications of transurethral resection of the prostate? | Transurethral resection of the prostate (TURP) is a common surgical procedure used to treat men with benign prostatic hyperplasia (BPH), a condition in which the prostate gland enlarges and obstructs the urethra. Although TURP is generally safe, like all surgical procedures, it carries potential risks and complications. Here are some of the potential complications of TURP:  TURP Syndrome (or Transurethral Resection (TUR) Syndrome): This is a rare but potentially serious complication caused by the absorption of irrigation fluid used during the procedure. This can lead to dilutional hyponatremia (low sodium in the blood), which can result in symptoms like nausea, vomiting, headache, confusion, high blood pressure, bradycardia (slow heart rate), seizures, and even coma.  Bleeding: Some amount of bleeding is expected, but excessive bleeding may require blood transfusion. It may also lead to blood clots in the bladder, which might need to be flushed out or resected.  Urinary Infections: The procedure might introduce bacteria into the urinary tract, leading to infections.  Urinary Incontinence: Some men might experience difficulty controlling urine after the procedure.  Erectile Dysfunction: Although TURP primarily affects the prostate and not the structures responsible for erection, some men might experience changes in their erectile function after the surgery.  Retrograde Ejaculation: This is one of the most common side effects of TURP. It happens when semen flows backward into the bladder instead of out through the urethra during ejaculation. It doesn't harm the body but can affect fertility.  Urethral Stricture: Scarring of the urethra can cause narrowing (stricture), leading to difficulties in passing urine.  Bladder Neck Contracture: Scarring at the junction of the bladder and prostate can cause narrowing, leading to urinary symptoms.  Urinary Retention: Some patients might have difficulty urinating after the procedure and may require catheterization for a period.  Perforation of the Prostate: Rarely, the surgical instrument might create a hole in the prostate capsule, leading to complications.  Death: As with any surgery, there is a small risk of death associated with anesthesia and the procedure itself.  Postoperative pain: This is common after the procedure but typically subsides with time and pain medications.  It's essential for patients to discuss potential risks and benefits of TURP with their urologists before undergoing the procedure. Proper postoperative care, monitoring, and follow-up with the healthcare provider can help manage and mitigate many of these potential complications. | *EAU patient portal*:  You will usually be advised to avoid sexual activity for 2-3 weeks and it is not uncommon when you do to experience what is called “retrograde ejaculation”. Sometimes called a dry orgasm, retrograde ejaculation is a condition where semen goes into the bladder, rather than through your urethra and out of your penis during an orgasm. It isn’t harmful and the semen will pass through the bladder when you pee, but it can cause fertility issues.  If you have undergone TURP surgery and are producing little to no semen when you climax, have cloudy urine after sex, or your female partner is struggling to get pregnant, then you may wish to talk to your doctor about a test for retrograde ejaculation.  *AUA patient portal*:  After surgery, it can take time for sexual function to return fully. Most experts believe that if you were able to have an erection shortly before surgery, you will be able to after surgery. Your orgasm is not expected to change. In some cases, men can experience an issue where semen enters the bladder rather than out the penis (retrograde ejaculation.) Infection, bleeding and incontinence may also occur after some BPH treatments. In some cases, scar tissue may form. Side effects vary with the type of treatment you choose and most side effects are temporary. Some men need further or new treatment for their BPH symptoms after initial treatment. It is of great value to tell your doctor about any side effect you may have to get help. |
|  | 46. What is the efficacy of transurethral resection of the prostate? | Transurethral resection of the prostate, or TURP, is a procedure done to help men who have trouble urinating due to an enlarged prostate. Here's a basic rundown of what it involves and its benefits:  What is TURP?  It's a surgery where the doctor removes a portion of the prostate gland that's causing urinary problems. The procedure is done through the urethra, the tube that carries urine out of your body, so there are no external cuts.  Why might you need it?  As some men get older, their prostate can grow and press on the tube that lets urine flow out. This can make it hard to start peeing, cause a weak stream, or make you feel like you still have to go even after you've finished.  How effective is it?  Most men who have the procedure notice a big improvement in their symptoms. They can pee more easily and empty their bladder better.  Is it better than medications?  There are medicines for this condition, and they can help. But TURP usually provides more significant relief and works right away, while medicines might take time to show their full effect.  Will it improve my life?  Many men say the procedure makes their day-to-day life better since they don't have to worry as much about finding a bathroom urgently or waking up multiple times at night to pee.  Are there any side effects?  As with any surgery, there can be risks. Some men might have bleeding or get an infection. One common side effect is called "retrograde ejaculation," where during sexual activity, the semen goes into the bladder instead of coming out. It's not harmful, but it might feel different.  Are there other options?  Yes, there are newer methods too, like using lasers or other devices. Your doctor will talk to you about the best option for your specific situation.  Remember, the decision to have TURP or any procedure is yours. It's essential to discuss any concerns with your doctor and get all your questions answered.  I hope that makes things clearer! If you have any more questions or need further clarification, don't hesitate to ask. | No information available in EAU and AUA patient portals |
|  | 47. What is the recurrence of benign prostatic enlargement after transurethral resection of the prostate? | Imagine your prostate is like a small walnut-shaped gland that sits around a part of your urine tube. As some men get older, this gland can grow larger. This is what we call benign prostatic enlargement, or BPE. It's not cancer, but it can press on the tube and make it hard for you to pee.  To help with this, we sometimes do a procedure called TURP. This is where we carefully remove some of the enlarged prostate to make it easier for you to pee. It's a common and effective treatment.  Now, there's something we need to consider. Even after this procedure, there's a small chance that the prostate might start to grow again over time. This doesn't happen quickly, usually taking many years, but it's something we keep an eye on.  If your prostate does start to grow again, you might notice some of the old symptoms coming back, like trouble peeing. But don't worry, we'll be keeping a close check on you with regular visits and tests. If there's any sign of the prostate growing again, we have many ways to manage it, from medicines to other treatments.  Remember, every person is different, and we'll make sure to find the best way to keep you comfortable and healthy. | *EAU patient portal*:  BPE cannot be completely cured and all medical and surgical treatments that are available focus on reducing the symptoms as far as possible. BPE symptoms and how bothersome they are can change over time. You may or may not decide to have further or more invasive treatment depending on your circumstances or how much the symptoms affect your daily life.  Your doctor will help you to make informed decisions about how you decide to treat your BPE and advise you of any new treatment options available so that your BPE is as well-managed as it can be, and you are able to enjoy your relationships and participate in all aspects of your life.  *AUA patient portal*:  No information available |
|  | 48. What is a greenlight laserTM photovaporization of the prostate? | GreenLight Laser Therapy is a treatment used when the prostate gland gets bigger, causing problems with passing urine. This growth is not cancer; it's just something that happens to some men as they get older.  How does it work?  Imagine the prostate as a donut blocking a water pipe (your urethra). This laser acts like a precise, controlled heat source that "melts away" the extra part of the donut, making more room in the water pipe.  Why use this laser?  Quick Relief: Many men notice they can pee better quite soon after the procedure.  Short Hospital Stay: You might be able to go home the same day.  Less Bleeding: The laser is gentle and reduces the chance of bleeding during the procedure.  Fast Recovery: You can get back to your regular activities sooner than with some other treatments.  Are there any side effects?  Like all treatments, there can be side effects. Some men might get a urinary infection, feel a burning sensation when peeing, or notice changes in how they ejaculate. It's essential to chat with your doctor about these so you're informed and prepared.  In a nutshell: This therapy uses a special laser to carefully remove the excess part of your prostate, helping you pee more easily. It's a modern method with many benefits, but always discuss with your doctor to see if it's right for you. | *EAU patient portal*:  A thin device called a resectoscope is inserted through the urethra. The resectoscope has a laser at the end of it which uses heat to destroy part of the prostate tissue, and a camera that allows the surgeon to see a high-quality image on a video monitor. The procedure is done under a general anaesthetic or spinal anaesthetic so you will not feel anything.  *AUA patient portal*:  Photoselective vaporization (PVP) is a way to vaporize prostate tissue with a high-powered laser. Most men can have a PVP without problems. The procedure is done as an outpatient procedure at the hospital or sometimes in the doctor’s office. |
|  | 49. What are the complications of a greenlight laserTM photovaporization of the prostate? | Hello! So, you're considering the Green laser treatment for your prostate enlargement. It's a popular choice because it's less invasive and has a quicker recovery time than some other procedures. But like all medical procedures, it's essential to know the potential side effects and complications. Let me break them down for you:  Blood in the urine: After the procedure, you might see a bit of blood when you urinate. It's normal and usually clears up in a few days.  Urinary discomfort: You may feel a strong urge to urinate, need to go more often, or find it a bit painful. These feelings generally get better as you heal.  Urinary infection: Any procedure involving the urinary system can sometimes lead to an infection. If you have symptoms like burning when you pee or fever, let us know.  Change in ejaculation: Some men find that when they ejaculate, the semen goes backward into the bladder instead of out. It's harmless but could affect fertility.  Leaking urine: It's rare, but some men might have a bit of urine leakage after the procedure.  Narrowing of the urine passage: Sometimes, after healing, the area where we did the procedure can narrow due to scar tissue, making it hard to pee.  Penetration of the laser: In rare cases, the laser might go deeper than we intend. If this happens, it could lead to bleeding.  Fluid absorption: This is very rare with the Green laser, but sometimes the body might take in too much of the fluid we use during the surgery. This can lead to an imbalance in the body.  Erection changes: A few men notice changes in their ability to have or keep an erection after the treatment.  Symptoms might not go away: Even after the procedure, some men might still have urinary symptoms.  Bladder neck narrowing: Rarely, the entrance to the bladder can narrow due to scarring, causing some urinary symptoms.  Remember, while these potential side effects and complications exist, many men go through this procedure without any issues. We'll monitor your recovery closely, and you should always reach out if you notice anything unusual or if you have any concerns. The goal is to make you feel better and improve your quality of life. Let's work together to achieve that! | *EAU patient portal*:  You will usually be advised to avoid sexual activity for 2-3 weeks and it is not uncommon when you do to experience retrograde ejaculation. Sometimes called a dry orgasm, retrograde ejaculation is a condition where semen goes into the bladder, rather than through your urethra and out of your penis during an orgasm. It isn’t harmful and the semen will pass through the bladder when you pee, but it can cause fertility issues.  If you have had laser vaporisation and are producing little to no semen when you climax, have cloudy urine after sex, or your female partner is struggling to get pregnant, then you may wish to talk to your doctor about a test for retrograde ejaculation.  *AUA patient portal*:  There are few side effects. After PVP, most men can stop medical therapy. This method is useful for most men except those with the largest of prostates. Because this method causes very little blood loss it is particularly useful in men with a higher risk of bleeding, such as those taking blood-thinning medications or those with weak hearts. |
|  | 50. What is the efficacy of greenlight laserTM photovaporization of the prostate? | GreenLight Laser Therapy is a treatment for an enlarged prostate, a condition we also call Benign Prostatic Hyperplasia or BPH. In this treatment, a special green laser is used to remove the extra prostate tissue that's causing urinary problems.  Why might you consider it?  Symptom Relief: Many men find that after this treatment, their urinary problems improve a lot. They can pee more easily and don't have to get up as many times during the night.  Quick Recovery: It's a procedure that doesn't involve major surgery. Most men can go home the same day or the next day and get back to their regular activities within a few days.  Safety: This treatment is considered safe, even for men taking blood thinners.  Less Blood Loss: Compared to other prostate treatments, there's usually very little bleeding with the GreenLight laser.  Are there any downsides?  Might Need Another Treatment Later: Some men might need to have the treatment again in the future, but this isn't very common.  Experience Matters: The results might be better if the doctor has done this procedure many times before. So, it's a good idea to ask your doctor about their experience.  In simple terms, the GreenLight Laser Therapy is like a modern tool to help with prostate problems. It has many benefits, but like any medical treatment, it's essential to discuss with your doctor if it's the right choice for you. | No information available in EAU and AUA patient portals |
|  | 51. What is the recurrence of benign prostatic enlargement after greenlight laserTM photovaporization of the prostate? | Certainly! When we talk about GreenLight laser therapy for treating benign prostatic enlargement (BPE), it's a procedure where a special kind of laser is used to reduce the size of the enlarged prostate. This helps in relieving symptoms like difficulty in urinating.  Now, when it comes to how often patients need to undergo this procedure again, the good news is that the chances of needing another surgery are quite low. In a detailed study, after following up with patients for about 57 months, which is almost 5 years, only 4.8% of them needed to have the surgery again. This means that for the vast majority of patients, the benefits of the first GreenLight laser treatment lasted for this period without needing another operation.  So, in simpler terms, if 100 people had this treatment, about 5 might need to have the procedure again within five years, but around 95 would not. This is a positive outcome, showing that the treatment is effective for most people in the long term. | *EAU patient portal*:  BPE cannot be completely cured and all medical and surgical treatments that are available focus on reducing the symptoms as far as possible. BPE symptoms and how bothersome they are can change over time. You may or may not decide to have further or more invasive treatment depending on your circumstances or how much the symptoms affect your daily life.  Your doctor will help you to make informed decisions about how you decide to treat your BPE and advise you of any new treatment options available so that your BPE is as well-managed as it can be, and you are able to enjoy your relationships and participate in all aspects of your life.  *AUA patient portal*:  No information available |
|  | 52. What is a Holmium laser enucleation of the prostate? | Hello! So, you're here to learn about the Holmium laser enucleation of the prostate, or HoLEP for short. Let me explain it in simple terms.  Your prostate is a small gland located below the bladder. Sometimes, as men get older, this gland can grow bigger than it should. This growth is not cancer, but it can press on the tube you pee through (the urethra) and cause problems with urination.  HoLEP is a procedure we use to help with this issue. Here's how it works:  Getting to the Prostate: First, you'll be given something to make sure you're comfortable and don't feel pain – like being put to sleep or numbing the lower part of your body. We then use a special instrument that we can look through, and we gently insert it into the urethra.  Making a Cut and Removing the Extra Growth: Once we can see the prostate clearly, we use a laser (a concentrated light beam) to make a cut and remove the part of the prostate that's grown too much. This laser is great because it can cut and also stop any small bleeds at the same time.  Clearing Out the Removed Tissue: After removing the extra prostate tissue, the pieces go into the bladder. We use another tool to break these pieces into smaller bits and then remove them.  Finishing Up: Once everything's cleared out, we remove our instruments, and that's pretty much it!  The benefits of this procedure include:  No Cuts on the Outside: Everything is done from the inside, through the urethra.  Quick Recovery: Most people get better fast and can go home soon after the procedure.  Lasting Results: The benefits of the surgery, like easier peeing, tend to last a long time.  Like all procedures, there might be some side effects, like infection or some temporary issues with peeing. But these are generally rare, especially if the doctor is very experienced.  It's important to talk with your doctor about this procedure to see if it's right for you. They'll be able to answer all your questions and help you decide. | *EAU patient portal*:  Laser enucleation of the prostate (also called Holmium laser enucleation of the prostate or HoLEP)  Laser enucleation is generally suitable for men who have an enlarged prostate over a certain size (usually 80 mL, approximately 4 times the size of a healthy prostate). The procedure involves a surgeon passing a thin device called a resectoscope through the urethra. The resectoscope has a high pulse laser at the end of it which removes the prostate gland and a camera that allows the surgeon to see a high-quality image on a video monitor. The prostate is cut into very small pieces and removed using a surgical tool passed through the urethra.  The procedure is done under a general anaesthetic or spinal anaesthetic so you will not feel anything.  *AUA patient portal*:  Holmium laser enucleation of prostate (HoLEP) is when a surgeon places a resectoscope through the penis into the urethra. A laser inserted into the resectoscope destroys excess prostate tissue. No incisions (cuts) are needed and there is very little bleeding. You may only need to stay one night in the hospital. |
|  | 53. What are the complications of a Holmium laser enucleation of the prostate? | Hello! I understand you're considering or are scheduled for a procedure called HoLEP to help with an enlarged prostate. It's a modern method that uses a laser to remove excess prostate tissue and has been found to be effective for many men. But, like any medical procedure, there are potential risks and side effects. Here's what you should be aware of:  Urinary Infections: Sometimes, due to the tools we use, there's a chance you might get a urinary infection. This can be treated with antibiotics.  Blood in the Urine: It's common to see a bit of blood in the urine after surgery, but this usually goes away on its own in a few days.  Temporary Leakage: Some men experience a bit of urine leakage after the surgery. This usually gets better over time.  Changes in Ejaculation: After the procedure, you might notice that during climax, the semen doesn't come out the usual way. Instead, it goes into the bladder. This doesn't harm you, but it can affect fathering a child.  Narrowing of the Urine Passage: Rarely, scar tissue can develop, making the urine passage narrower. If this happens, we might need to treat it.  Scar Tissue at the Bladder Entry: Sometimes, scar tissue can form where the bladder and prostate meet, which might require treatment.  Fluid Absorption: Very rarely, the body might absorb some of the fluid we use during the procedure, which can lead to symptoms like confusion or nausea. We monitor for this closely.  Painful Urination: Some men feel a bit of discomfort when urinating after the surgery, but this usually improves with time.  Injuries: There's a very small chance of injury to nearby parts like the bladder, but this is rare.  Need for Another Surgery: In rare cases, we might need to do another procedure if some prostate tissue remains or if there are complications.  I want you to be aware of these potential risks, but please know that many men undergo this procedure without complications. The decision to have this surgery should be based on your needs and after a full discussion with your doctor about the benefits and risks. If you have any questions or concerns, please don't hesitate to ask! | *EAU patient portal*:  You will usually be advised to avoid sexual activity for 2-3 weeks and it is not uncommon when you do to experience retrograde ejaculation. Sometimes called a dry orgasm, retrograde ejaculation is a condition where semen goes into the bladder, rather than through your urethra and out of your penis during an orgasm. It isn’t harmful and the semen will pass through the bladder when you pee, but it can cause fertility issues.  If you have undergone laser enucleation surgery and are producing little to no semen when you climax, have cloudy urine after sex, or your female partner is struggling to get pregnant, then you may wish to talk to your doctor about a test for retrograde ejaculation.  *AUA patient portal*:  After surgery, it can take time for sexual function to return fully. Most experts believe that if you were able to have an erection shortly before surgery, you will be able to after surgery. Your orgasm is not expected to change. In some cases, men can experience an issue where semen enters the bladder rather than out the penis (retrograde ejaculation.) Infection, bleeding and incontinence may also occur after some BPH treatments. In some cases, scar tissue may form. Side effects vary with the type of treatment you choose and most side effects are temporary. Some men need further or new treatment for their BPH symptoms after initial treatment. It is of great value to tell your doctor about any side effect you may have to get help. |
|  | 54. What is the efficacy of a Holmium laser enucleation of the prostate? | It's a procedure to treat an enlarged prostate, which can cause problems like slow urine flow or frequent trips to the bathroom. Instead of making large cuts, we use a special laser to remove the extra prostate tissue that's causing trouble.  How does it compare to other treatments?  Many studies show that HoLEP provides excellent relief from symptoms. Some even find it better than the more common surgery called TURP. Especially for men with a really large prostate, HoLEP can be a great choice.  Benefits of HoLEP:  Less Blood Loss: There's typically less bleeding during this procedure compared to others.  Short Hospital Stay: Most people can go home the next day.  Quick Recovery: You can get back to your normal routine relatively fast.  Lasting Results: The chances of needing another surgery in the future are quite low.  Sexual Health: There's a low risk of problems with sexual function after this procedure. Though there's a chance that during ejaculation, semen might flow backward into the bladder. This doesn't harm your health, but it can change the experience.  Safety: Like any surgery, there are some risks, like blood in the urine or infections. But serious problems are rare.  Surgeon's Experience Matters: Not all doctors perform this procedure often, so it's essential to choose someone experienced.  In short, HoLEP is a modern, efficient way to treat an enlarged prostate, especially if it's really big. It's less invasive, which means quicker recovery for you. But always remember, it's crucial to discuss with your doctor to see if it's the best option for your specific situation. | No information available in EAU and AUA patient portals |
|  | 55. What is the recurrence of benign prostatic enlargement after Holmium laser enucleation of the prostate? | Holmium Laser Enucleation of the Prostate (HoLEP) is a surgical procedure used to treat benign prostatic enlargement (BPE), a common condition in older men. The good news is that this procedure has a high success rate and a low chance of needing a repeat surgery. Studies show that about 95% of patients don't require another operation for at least 10 years after their HoLEP procedure.  In some cases, there might be a need for additional treatment, but this is relatively rare. Factors like the size of your prostate before the surgery, how much your PSA levels decrease after the procedure, and whether you've had prostate surgery before, can influence your chances of needing further treatment. Overall, HoLEP is considered a very effective and long-lasting treatment for BPE. | *EAU patient portal*:  BPE cannot be completely cured and all medical and surgical treatments that are available focus on reducing the symptoms as far as possible. BPE symptoms and how bothersome they are can change over time. You may or may not decide to have further or more invasive treatment depending on your circumstances or how much the symptoms affect your daily life.  Your doctor will help you to make informed decisions about how you decide to treat your BPE and advise you of any new treatment options available so that your BPE is as well-managed as it can be, and you are able to enjoy your relationships and participate in all aspects of your life.  *AUA patient portal*:  No information available |
|  | 56. What is a Thulium laser enucleation of the prostate? | Hey there! So, you're here to learn about the Thulium laser procedure for an enlarged prostate, right? Let's break it down step-by-step:  The Problem: Sometimes, as men get older, their prostate (a small gland below the bladder) can get bigger. This can make it hard to urinate normally.  The Solution: One way to treat this is with a procedure called Thulium laser enucleation of the prostate, or "ThuLEP" for short.  What Happens During the Procedure?:  You'll be given something so you won't feel any pain, like being put to sleep or numbing the area.  A doctor will then insert a tiny camera into the urinary pipe (the tube you pee from) to see the prostate.  Using a special laser, the doctor will carefully remove the parts of the prostate that are causing the blockage.  This unwanted tissue is then pushed into the bladder, chopped into tiny bits, and removed.  Why Use This Laser Technique?:  It causes very little bleeding.  You can recover quickly and might be able to go home the next day!  It's effective in helping many men pee more easily.  Are There Any Risks?: Like any procedure, there are some risks. Rarely, there might be an infection or some issues with urination. Some men might also notice a change in the way they feel when they ejaculate, but it's not harmful.  After the Procedure: You might have a small tube (called a catheter) in your urinary pipe for a short while to help you pee. You'll be advised to take it easy, not do any heavy lifting, and you might be given some medicines to help with any discomfort.  I hope that makes things clearer! If you have any concerns or questions, always feel free to ask your doctor. They're there to help! | *EAU patient portal*:  No information available  *AUA patient portal*:  Thulium laser enucleation of the prostate (ThuLEP) is similar to HoLEP but uses a different type of laser. As in HoLEP, the surgeon places a resectoscope through the penis into the urethra. A laser inserted into the resectoscope destroys excess prostate tissue. Men with larger prostates who wish to avoid more-invasive surgery may be good candidates for this treatment. No incisions (cuts) are needed and there is very little bleeding. |
|  | 57. What are the complications of a Thulium laser enucleation of the prostate? | Thulium laser enucleation of the prostate (or ThuLEP for short) is a procedure we use to treat an enlarged prostate. This enlargement can cause problems with urination. During the procedure, we use a special laser to remove the excess prostate tissue that's causing the blockage.  Like any procedure, there are potential side effects and complications, but I want to assure you that they are generally rare. Here's what you might expect:  Blood in the urine: After the procedure, you might notice some blood in your urine. This is common and should clear up on its own in a few days or weeks.  Infection: As with any procedure in the urinary system, there's a small chance of getting an infection. We'll give you guidelines on how to reduce this risk.  Trouble urinating: Some men have difficulty urinating after the surgery. If this happens, we might need to use a small tube (catheter) temporarily to help you pee.  Change in ejaculation: Some men notice that during sexual activity, the semen goes backward into the bladder instead of coming out. This doesn't harm you or affect the pleasure but might feel different.  Narrowing of the urinary tube: Rarely, scarring can cause the tube you pee through (urethra) to become narrow, which might affect urination.  Burning sensation when peeing: Some men might feel a burning sensation when they pee. This typically goes away with time.  Sudden urge to pee: You might occasionally feel a sudden strong need to pee and might leak some urine. This usually improves over time.  Erection changes: It's rare, but some men might experience changes in their ability to get or maintain an erection after the procedure.  I'll be here to guide you through the process and answer any questions you might have. Remember, this procedure is done to help alleviate the symptoms you're experiencing from your enlarged prostate, and many men have benefitted from it. We'll work together to ensure the best possible outcome for you! | *EAU patient portal*:  No information available  *AUA patient portal*:  After surgery, it can take time for sexual function to return fully. Most experts believe that if you were able to have an erection shortly before surgery, you will be able to after surgery. Your orgasm is not expected to change. In some cases, men can experience an issue where semen enters the bladder rather than out the penis (retrograde ejaculation.) Infection, bleeding and incontinence may also occur after some BPH treatments. In some cases, scar tissue may form. Side effects vary with the type of treatment you choose and most side effects are temporary. Some men need further or new treatment for their BPH symptoms after initial treatment. It is of great value to tell your doctor about any side effect you may have to get help. |
|  | 58. What is the efficacy of a Thulium laser enucleation of the prostate? | Hello! I understand you have some questions about a procedure called Thulium laser enucleation of the prostate, or ThuLEP for short. Let's break it down:  What is ThuLEP?  It's a modern surgical method to treat an enlarged prostate, a condition many older men face. In this procedure, we use a Thulium laser to gently and precisely remove the part of the prostate that's causing problems.  Why might you consider ThuLEP?  Relief from Symptoms: Many men who have this procedure experience significant relief from bothersome urinary symptoms.  Quick Recovery: Compared to some traditional surgeries, recovery is often faster, and you may spend less time in the hospital.  Low Chances of Repeating the Procedure: The effects of this procedure are long-lasting, meaning there's a low chance you'll need another operation later on.  Safety: It's generally a safe procedure with a few typical side effects, similar to other prostate surgeries. Severe complications are rare.  Possible Side Effects?  Like any surgery, there might be some side effects like urinary tract infections or temporary changes in urinary habits. But these issues are typically manageable and temporary.  Is it suitable for everyone?  Mostly yes. This technique works well for many men with different prostate sizes. But as with any medical treatment, it's essential to have a detailed discussion about what's best for you.  To sum it up, ThuLEP is a modern, effective, and safe way to treat an enlarged prostate. If you're considering it, we can chat further about the details and ensure it's the right choice for you.  Remember, it's always a good idea to discuss any concerns or questions you have with your doctor. They'll provide guidance tailored to your specific situation. | No information available in EAU and AUA patient portals |
|  | 59. What is the recurrence of benign prostatic enlargement after Thulium laser enucleation of the prostate? | Benign Prostatic Enlargement, or BPE, is when the prostate gland gets larger, which is quite common as men get older. You've undergone a procedure called ThuLEP, which is a laser treatment to remove a portion of the prostate and relieve your symptoms.  The good news is that after this procedure, the chances of your BPE coming back are generally low. ThuLEP is known to be effective because it removes a significant amount of the prostate tissue, which means there's less chance for the prostate to grow large again soon.  Of course, every person is different. The risk of the BPE returning can depend on several factors, such as how big your prostate was before the surgery and your overall health. But in most cases, patients experience a long-lasting relief from their symptoms and don't need any more treatment for a long time.  It's important to keep up with regular check-ups after your surgery. This way, we can keep an eye on your prostate and make sure everything is going well. If there are any changes or if you have any concerns, we'll be able to address them promptly."  This explanation is meant to be reassuring and informative, emphasizing the effectiveness of ThuLEP while also encouraging ongoing health monitoring. | *EAU patient portal*:  BPE cannot be completely cured and all medical and surgical treatments that are available focus on reducing the symptoms as far as possible. BPE symptoms and how bothersome they are can change over time. You may or may not decide to have further or more invasive treatment depending on your circumstances or how much the symptoms affect your daily life.  Your doctor will help you to make informed decisions about how you decide to treat your BPE and advise you of any new treatment options available so that your BPE is as well-managed as it can be, and you are able to enjoy your relationships and participate in all aspects of your life.  *AUA patient portal*:  No information available |
|  | 60. What is aquablation for benign prostatic enlargement? | Aquablation is a modern treatment for an enlarged prostate, a condition many older men face. This enlargement can cause problems with passing urine.  How Does It Work?  Imagine your enlarged prostate is like an overgrown hedge blocking a pathway. Aquablation uses a thin, controlled stream of water (like a mini water-jet) to trim away the extra growth, clearing the pathway. This is all done with the help of a special camera, so the doctor can see exactly what they're doing.  Why Choose Aquablation?  Gentle: Unlike some other treatments, Aquablation doesn't use heat, so there's less chance of unwanted side effects from burns.  Accurate: The water-jet is controlled by a robot, making it very precise. It trims away the extra growth without harming the essential parts of the prostate.  Quick Recovery: Many men find relief from their symptoms soon after the procedure.  Good for Sexual Health: Early studies show that men might have fewer sexual side effects with this treatment compared to some other methods.  Is It Right for Me?  Every man is different. It's crucial to talk with your doctor about the pros and cons of Aquablation and see if it's a good fit for your health and lifestyle. | *EAU patient portal*:  This is a new treatment for BPE. Water is injected into the prostate using a probe passed into the urethra. The pressure of the water is used to destroy some of the prostate tissue, making it smaller.  Water ablation is less likely to cause side effects, however not all surgeons are trained to do this procedure.  *AUA patient portal*:  Transurethral water–jet ablation (TWJA) uses highpressure water jets to destroy excess prostate tissue. The surgeon first uses ultrasound to precisely map the location of the excess tissue. Then the high-pressure water jets are directed to that area. Following this, the surgeon inserts another instrument to seal small blood vessels to reduce the risk of bleeding. The patient needs to stay in the hospital one night to irrigate the bladder to prevent blood clots. |
|  | 61. What are the complications of aquablation for benign prostatic enlargement? | Aquablation is a newer method we use to treat an enlarged prostate, a common condition that can cause difficulty with urination. Instead of traditional surgery, we use a stream of water to clear away the extra prostate tissue that's causing the blockage.  Like any procedure, there are some possible side effects and complications. Let's go over them:  Blood in the Urine: After the procedure, you might notice some blood in your urine. This is common and usually clears up on its own after a little while.  Urinary Infections: Sometimes, procedures involving the bladder can lead to infections. If you experience symptoms like burning when you pee or fever, let us know.  Difficulty Urinating: Some people find it hard to urinate right after the procedure. If that happens, we might need to use a small tube called a catheter for a short time to help.  Leakage of Urine: In some cases, you might have some unexpected urine leaks. This doesn't happen to everyone, but it's something to be aware of.  Changes in Ejaculation: After the procedure, some men find that during climax, the semen goes backward into the bladder instead of coming out. This doesn't hurt and you won't feel much different, but it's a change some people notice.  Narrowing of the Urine Passage: Rarely, scar tissue can form and narrow the passage where urine flows out. If that happens, we have ways to fix it.  Bladder Injury: It's rare, but there's a small chance of injury to the bladder. We'll be monitoring for this.  Not a Complete Fix: Even after the procedure, some people might still have symptoms. If that happens, we'll discuss the next steps.  Changes in Erection: Some men might notice changes in their ability to have an erection. This isn't common but is a possibility.  Some Pain or Discomfort: It's normal to feel a bit of discomfort after the procedure. We'll give you guidance on how to manage it.  Need for Another Treatment: Just like other treatments for an enlarged prostate, there's a chance that in the future, you might need another procedure.  Remember, our goal is to improve your quality of life and ease those urinary symptoms. We're here to answer any questions and address any concerns you might have! | *EAU patient portal*:  No information available  *AUA patient portal*:  After surgery, it can take time for sexual function to return fully. Most experts believe that if you were able to have an erection shortly before surgery, you will be able to after surgery. Your orgasm is not expected to change. In some cases, men can experience an issue where semen enters the bladder rather than out the penis (retrograde ejaculation.) Infection, bleeding and incontinence may also occur after some BPH treatments. In some cases, scar tissue may form. Side effects vary with the type of treatment you choose and most side effects are temporary. Some men need further or new treatment for their BPH symptoms after initial treatment. It is of great value to tell your doctor about any side effect you may have to get help. |
|  | 62. What is the efficacy of aquablation for benign prostatic enlargement? | Hey there!  So, you've been hearing about this treatment called "Aquablation" for an enlarged prostate, right? Let me break it down for you:  What's Aquablation? It's a newer way to treat an enlarged prostate, which can cause difficulties in urinating. In this method, a robot-controlled waterjet is used to remove the excess prostate tissue that's causing the problem.  Does It Work? Yes, the studies so far are promising. Most men who have had the procedure experience fewer urinary problems afterward.  Is It Safe? From what we know now, it seems to have a good safety record. Every procedure has risks, but the serious problems with this one are relatively rare.  Any Sexual Side Effects? One of the good things about Aquablation is that it seems to have a lower chance of causing sexual problems compared to some other treatments.  Does the Size of the Prostate Matter? Some treatments are less effective if the prostate is really large. However, Aquablation seems to work well even for those with bigger prostates.  If you're thinking about this treatment, it's essential to chat with a urologist. They'll give you personalized advice based on your specific situation. | No information available in EAU and AUA patient portals |
|  | 63. What is the recurrence of benign prostatic enlargement after aquablation? | Aquablation is a newer, less invasive surgery to treat an enlarged prostate, a common condition in older men. Instead of cutting, it uses a water jet to remove the extra prostate tissue.  Does the Enlargement Come Back After the Surgery?: It's possible, but early signs are good. Aquablation seems to work well at reducing symptoms with a lower chance of needing another treatment compared to older surgeries. But, it's a relatively new method, so doctors are still learning about how it works in the long term.  What Affects Whether the Enlargement Comes Back?: Several things can play a role, like how old you are, the size of your prostate, how much tissue is removed during surgery, and your overall health.  Follow-Up Care is Important: After you have Aquablation, it's important to keep up with your doctor appointments. They will check to make sure your symptoms aren’t coming back and that there are no complications from the surgery.  Comparing with Other Treatments: Every treatment for an enlarged prostate has its own pros and cons, including how likely it is for symptoms to come back. Your doctor can help you understand how Aquablation compares with other options.  In short, Aquablation is a promising new way to treat an enlarged prostate with some advantages, but like any medical treatment, it's important to discuss with your doctor about what's best for your specific situation. | *EAU patient portal*:  BPE cannot be completely cured and all medical and surgical treatments that are available focus on reducing the symptoms as far as possible. BPE symptoms and how bothersome they are can change over time. You may or may not decide to have further or more invasive treatment depending on your circumstances or how much the symptoms affect your daily life.  Your doctor will help you to make informed decisions about how you decide to treat your BPE and advise you of any new treatment options available so that your BPE is as well-managed as it can be, and you are able to enjoy your relationships and participate in all aspects of your life.  *AUA patient portal*:  No information available |
|  | 64. What is a simple prostatectomy? | What is a simple prostatectomy for BPE?  Imagine your prostate as a doughnut-shaped gland surrounding a tube (the urethra) that you pee through. As men get older, this gland can get bigger, squeezing that tube and making it harder to pee. This growing gland is called "Benign Prostatic Enlargement" or BPE. "Benign" means it's not cancer.  Now, think of a simple prostatectomy as a procedure where a doctor removes the inner part of that doughnut, leaving the outer ring. This makes more space for the pee to flow out.  How is it done?  Going to Sleep: The doctor will give you medicine so that you either sleep through the procedure or feel no pain.  Making an Opening: The doctor makes a small cut in your lower belly to reach the prostate.  Taking Out the Inside of the Doughnut: The doctor removes the inner part of your prostate that's squeezing on your pee tube. This will make peeing easier.  Finishing Up: After that's done, the doctor stitches everything back up and puts in a small tube (called a catheter) to help you pee while you heal.  Recovery: You'll stay in the hospital for a few days. The tube will be removed later when the doctor thinks you're healing well.  Remember, this procedure is just one of several ways to help men who have trouble peeing because of BPE. It's mostly used for men with a really big prostate. If you or someone you know is considering this, it's important to talk with the doctor about what's best for you, and to understand all the options available. | *EAU patient portal*:  open prostatectomy is only carried out in certain situations. This is because, while the surgery is known to be effective, it is major surgery and may take longer to heal. It is generally only suitable for men who have an enlarged prostate over a certain size (usually 80 mL, which is approximately 4 times the size of a healthy prostate).  During an open prostatectomy the prostate gland is removed through a cut in your lower abdomen under a general anaesthetic or spinal anaesthetic so you will either be asleep during the procedure or awake but will be numb from the waist down.  At the end of the operation, a catheter is placed in your bladder to help drain urine while you recover from the surgery.  *AUA patient portal*:  Simple prostatectomy removes the entire prostate gland with laparoscopic or robotic-assisted surgery. It is important for the surgeon to be skilled at this surgery. This is only offered to men with the largest of prostate glands. This is a long- term cure. |
|  | 65. What are the complications of a simple prostatectomy? | A simple prostatectomy is a surgery to address the enlargement of your prostate gland, which is a condition often seen in older men. This enlargement is benign, meaning it's not cancer. The goal of the surgery is to help you urinate more easily by removing the extra prostate tissue that's blocking the flow of urine.  Now, like all surgeries, there are some potential risks to be aware of:  Bleeding: It's possible for there to be some bleeding during or after the surgery. Rarely, a blood transfusion might be needed.  Infection: Sometimes, patients can get an infection in the urine or at the place where the surgery was done.  Leakage of urine: After surgery, some men might find it hard to hold in their urine and may leak a bit.  Trouble emptying the bladder: Even after the surgery, a few men might still find it hard to empty their bladder fully.  Narrowing of the urine tube: Scarring can occur, making the tube through which you pass urine narrower.  Scarring at the bladder opening: This is another form of scarring that can cause blockage.  Problems with erections: Some men might find it more challenging to get or keep an erection after the surgery.  Injury to nearby parts: There's a small chance of injury to areas near the prostate, like the rectum or bladder.  Blood clots: Being still for a long time after surgery can cause blood clots in the legs, and these can be dangerous if they travel to the lungs.  Fluid issues: The body might absorb too much fluid used during the surgery, leading to symptoms like nausea or high blood pressure.  Risks from anesthesia: Anesthesia is the medication that puts you to sleep during surgery. There are rare risks like allergic reactions or breathing problems.  Pain: You might feel some pain after surgery, but this is expected and can be managed.  Symptoms might come back: Over time, you might feel the same symptoms again, but this isn't common. | *EAU patient portal*:  You will usually be advised to avoid sexual activity for 2-3 weeks and it is not uncommon when you do to experience retrograde ejaculation. Sometimes called a dry orgasm, retrograde ejaculation is a condition where semen goes into the bladder, rather than through your urethra and out of your penis during an orgasm. It isn’t harmful and the semen will pass through the bladder when you pee, but it can cause fertility issues.  If you have undergone laser enucleation surgery and are producing little to no semen when you climax, have cloudy urine after sex, or your female partner is struggling to get pregnant, then you may wish to talk to your doctor about a test for retrograde ejaculation.  *AUA patient portal*:  After surgery, it can take time for sexual function to return fully. Most experts believe that if you were able to have an erection shortly before surgery, you will be able to after surgery. Your orgasm is not expected to change. In some cases, men can experience an issue where semen enters the bladder rather than out the penis (retrograde ejaculation.) Infection, bleeding and incontinence may also occur after some BPH treatments. In some cases, scar tissue may form. Side effects vary with the type of treatment you choose and most side effects are temporary. Some men need further or new treatment for their BPH symptoms after initial treatment. It is of great value to tell your doctor about any side effect you may have to get help. |
|  | 66. What is the efficacy of a simple prostatectomy for benign prostatic enlargement? | This is a treatment for an enlarged prostate, also known as benign prostatic hyperplasia or BPH.  What is it?  Imagine your prostate as an orange. The problem you're having is because the inside part of this "orange" has grown too big and is squeezing the tube (urethra) that you pee through. In this procedure, we remove that inside part, allowing urine to flow more easily. We'll leave the outer part intact.  Why might you need it?  If your prostate is very large, this procedure can really help in improving your symptoms. It's mainly for those whose prostate is bigger than what's typical for most BPH treatments.  What can you expect after the procedure?  Better Urine Flow: You should find it easier to pee, with a stronger and more consistent flow.  Fewer Symptoms: Issues like needing to pee often, especially at night, should improve.  Not Needing Further Treatment: Once this is done, most men don't need any more treatments for their enlarged prostate.  Are there risks?  Like all surgeries, there are some risks to be aware of:  Bleeding: Since the prostate has many small blood vessels, there's a chance of bleeding during the surgery.  Leaks: Some men might have a bit of trouble holding their pee afterward, but severe cases are rare.  Erection Changes: There's a small risk that the surgery might affect your ability to have an erection.  Infections: As with any surgery, there's a chance of getting an infection afterward.  Scar Tissue: Rarely, some men might develop scar tissue that can affect pee flow.  Recovery Time: You'll need to stay in the hospital a bit longer and might take some time to fully recover compared to other smaller procedures.  Final Thoughts:  This procedure can offer a lot of relief if you have a very enlarged prostate. It's important, though, to talk with your doctor about whether it's right for you and to understand both the benefits and risks. Always feel free to ask questions! | No information available in EAU and AUA patient portals |
|  | 67. What is the recurrence of benign prostatic enlargement after simple prostatectomy? | Hi there! You might be wondering about the chances of your symptoms coming back after your prostate surgery. Let me explain this:  What We Did in Surgery: In your surgery, we removed part of the prostate to help you pee more easily. We aimed to take out just the right amount to help with your symptoms.  How Often Symptoms Come Back: Most of the time, after this type of surgery, people don't have their symptoms come back. But, like with any surgery, there's a small chance it could happen.  What Affects the Chances of Symptoms Coming Back:  How the Surgery Went: We did our best to remove exactly what was needed. If we got it all, your symptoms are less likely to return.  Your Health: Your overall health plays a role. If you're generally healthy, that's a good sign for your recovery.  Taking Care After Surgery: Following our instructions after surgery, like taking it easy and keeping your follow-up appointments, helps lower the risk of symptoms coming back.  Regular Check-Ups: We'll keep an eye on things with regular visits. If anything looks like it's coming back, we'll catch it early.  Numbers and Statistics: Every person is different, so it's hard to give an exact number for the chances of symptoms returning. But in general, it's a low number for most people.  Remember, it's important to keep in touch with us and report any new symptoms or concerns. We're here to help and make sure you stay as healthy and comfortable as possible! | *EAU patient portal*:  BPE cannot be completely cured and all medical and surgical treatments that are available focus on reducing the symptoms as far as possible. BPE symptoms and how bothersome they are can change over time. You may or may not decide to have further or more invasive treatment depending on your circumstances or how much the symptoms affect your daily life.  Your doctor will help you to make informed decisions about how you decide to treat your BPE and advise you of any new treatment options available so that your BPE is as well-managed as it can be, and you are able to enjoy your relationships and participate in all aspects of your life.  *AUA patient portal*:  No information available |
| Minimally invasive techniques (MIST) | 68. What minimally invasive procedures are available for benign prostatic enlargement? | Alright, let's talk about the less-invasive treatments available for an enlarged prostate. Think of these as ways to relieve the pressure or size of your prostate without the need for major surgery:  UroLift: Imagine using tiny clips to pin back the parts of the prostate that are causing a blockage, kind of like pinning back curtains to let light in.  Rezūm Therapy: This is like using steam to gently shrink down the prostate. Over time, the steam makes the prostate smaller, helping you pee more easily.  GreenLight Laser Therapy: Think of this as a precise laser pointer melting away the extra bits of the prostate that are in the way.  HoLEP: This uses a laser to precisely cut away the extra prostate tissue. It's a bit more involved than the other laser method but can help with larger prostates.  Aquablation: Picture a tiny water jet washing away the extra bits of your prostate. It's guided by advanced imaging, so it's very precise.  TUMT: This uses gentle microwave heat to make parts of the prostate shrink down.  TUNA: We use tiny needles to deliver heat to specific spots in the prostate, causing those spots to shrink.  PAE: Imagine tiny beads blocking the blood flow to the parts of the prostate that are too big. This makes them shrink down over time.  Remember, the best treatment for you depends on your prostate's size, how it's affecting you, and other health factors. We'll discuss each option, weigh the benefits and risks, and decide together on the best approach for you. | *EAU patient portal*:  Aquablation of the prostate: A method used to reduce the size of the prostate using a water stream. This is done through the urethra, so no cuts are made in your belly.  Prostatic Artery Embolisation: A method used when there is an obstruction of the arteries that supply blood to the prostate. The procedure is done with specific tools that are introduced through an artery (blood vessel) in your arm or your leg.  Convective water vapour energy (WAVE) ablation, or the Rezum system  iTind  *AUA patient portal*:  Urolift  rezum  iTind  prostate artery embolization |
|  | 69. What is Urolift? | UroLift is a procedure designed to help men who have trouble urinating because of an enlarged prostate, a condition we sometimes call BPH.  How does it work?  Imagine your prostate is like a donut that has grown too big, squeezing the tube (the urethra) that you pee through. The UroLift procedure uses tiny implants to gently push apart the sides of that "donut", creating more space for urine to flow.  What can I expect during the procedure?  You might be given something to make you relaxed or sleepy.  A doctor will use a special device, which goes up the tube you pee through.  Tiny implants are placed to hold the prostate tissue apart.  The whole thing usually takes less than an hour.  Benefits of UroLift:  It doesn't involve cutting or removing any tissue.  Many men feel better quickly after the procedure.  You can usually go home the same day.  Are there side effects?  After the procedure, some men might feel a little discomfort, see some blood in their pee, or feel like they need to pee urgently for a short time. These effects usually don't last long.  Is it right for me?  If you're having trouble with urination due to an enlarged prostate, UroLift might be a good option. It's important to talk with your doctor about whether it's right for you and any questions you might have. | *EAU patient portal*:  During this procedure, a surgeon uses a thin device to insert implants into both sides of the prostate to force it away from the urethra, so it is no longer blocked. This helps to relieve symptoms such as pain or difficulty when urinating. It is usually done under sedation.  *AUA patient portal*:  uses a needle to place tiny  implants in the prostate. These implants lift and compress the enlarged prostate so that it no longer blocks the urethra. These implants stay in the body forever and are not easily removed. PUL may be done with either local or general anesthesia. |
|  | 70. What are the complications of Urolift? | Hello! I understand you're considering the UroLift procedure to help with your prostate-related urinary symptoms. Let me break down the procedure and the potential side effects for you.  UroLift is a procedure where we use tiny implants to lift the enlarged part of your prostate, so it doesn't press on your urine tube (urethra) anymore. This can help improve the flow of your urine.  However, like all medical procedures, there are some side effects to be aware of:  Feeling of Pain or Discomfort: After the procedure, you might feel some discomfort or pain down there, especially in the private parts.  Blood in Urine: You might notice your urine looking a bit pink or red. This is because of some blood, but it usually goes away in a few days.  Sudden Need to Urinate: You might feel the sudden urge to go to the bathroom more than usual.  Urinary Infection: Just like after any procedure, there's a small chance of getting a urinary tract infection, but we'll give you guidelines on how to reduce that risk.  Burning Sensation When Urinating: You might feel a burning sensation when you pee.  Trouble Urinating: Some men find it hard to pee after the procedure. If this happens, we might need to use a small tube (catheter) to help you out for a short time.  Pain in Lower Tummy: Some men feel pain in their lower belly area.  Implant Moving: In very rare cases, the tiny implant we put in might move a bit. If this happens, we'll address it.  Need for Another Procedure: Sometimes, if the UroLift doesn't help as much as we'd like, or if there's an issue, we might need to consider another treatment or procedure in the future.  I know this might sound like a lot, but remember, most men have a smooth experience with UroLift. It's essential to be informed about the possible side effects so you can make the best decision for your health. We're here to answer any questions and guide you through the process! | *EAU patient portal*:  No information available  *AUA patient portal*:  There are fewer sexual side effects with this, compared to other types of prostate surgery. MRI can still be done if you had a PUL. Talk  to your doctor about how PUL may impact the image quality of future MRI used for prostate cancer detection and if you are allergic to nickel, titanium or stainless steel. |
|  | 71. What is the efficacy of Urolift? | I'd like to explain a procedure called UroLift, which is one of the options for treating prostate enlargement or BPH, a common condition in men as they age. Here's what you should know:  What it Does: UroLift is a less invasive treatment that helps relieve urinary symptoms you might be experiencing due to your enlarged prostate.  Symptom Relief: Many men who have had the UroLift procedure notice an improvement in their urinary symptoms, like a reduced urge to urinate frequently or less difficulty in starting urination.  Quality of Life: This treatment can potentially improve your day-to-day comfort and reduce the bothersome symptoms related to BPH.  Recovery: One good thing about UroLift is that recovery is usually quicker compared to some other treatments. Most men can get back to their normal activities in just a few days.  Sexual Function: A great advantage of UroLift is that it typically doesn't impact sexual abilities, like some other treatments might.  Safety: Every procedure has some risks, but most men tolerate UroLift quite well. Some might experience temporary discomfort, like a burning feeling when urinating, but these usually go away on their own.  How Long It Lasts: The benefits of UroLift have been shown to last for several years for many men. However, everyone is different, and there's always a chance that some might need further treatment down the line.  In summary, UroLift is a treatment option that can offer relief from the symptoms of an enlarged prostate with a relatively quick recovery and fewer side effects compared to some other treatments. If you're considering it or have more questions, it's a good idea to chat with your doctor to see if it's right for you. | *EAU patient portal*:  No information available  *AUA patient portal*:  Current studies have evaluated five years of treatment with PUL and future studies may help to determine long term durability. |
|  | 72. What is the recurrence of benign prostatic enlargement after Urolift? | UroLift is a less invasive treatment for an enlarged prostate, a condition also known as benign prostatic hyperplasia (BPH). This condition can cause trouble with urination. The UroLift procedure helps by lifting and holding the enlarged prostate tissue so it no longer blocks the urethra (the tube through which urine exits the body).  How Does it Work? During the procedure, small implants are placed to hold the prostate lobes apart. This is done through a telescope inserted into the urethra, so there's no need for cutting or removing prostate tissue.  Recovery and Effectiveness: Recovery from UroLift is usually quicker than more invasive prostate surgeries. Most men find their urinary symptoms improve soon after the procedure. However, the results can vary from person to person.  Will the Symptoms Come Back? Over time, some men might find that their symptoms return. This can happen for a few reasons:  The prostate can keep growing, which might cause new blockage.  How well the procedure works can also depend on the shape and size of your prostate and how the implants were placed.  Everyone's body responds differently, so it's hard to predict exactly how long the benefits will last.  Follow-Up Care: Regular check-ups with your doctor are important. They can help manage any recurring symptoms and check on your overall prostate health.  What to Expect in the Long Term: UroLift is a relatively new procedure, so doctors are still learning about its long-term effectiveness. Many men have found lasting relief, but ongoing studies are helping us understand more.  Remember, it's always a good idea to talk openly with your doctor about any concerns or questions you have about your prostate health and treatment options. They can provide guidance tailored specifically to your health needs. | *EAU patient portal*:  BPE cannot be completely cured and all medical and surgical treatments that are available focus on reducing the symptoms as far as possible. BPE symptoms and how bothersome they are can change over time. You may or may not decide to have further or more invasive treatment depending on your circumstances or how much the symptoms affect your daily life.  Your doctor will help you to make informed decisions about how you decide to treat your BPE and advise you of any new treatment options available so that your BPE is as well-managed as it can be, and you are able to enjoy your relationships and participate in all aspects of your life.  *AUA patient portal*:  No information available |
|  | 73. What is REZUM? | Let me explain a treatment option called Rezūm, which we're considering for your enlarged prostate, a condition medically known as benign prostatic hyperplasia or BPH.  What is Rezūm?: Rezūm is a treatment that uses water vapor, or steam, to address the symptoms of an enlarged prostate. It's a less invasive option compared to some other treatments.  How does it work?: We use a device that sends tiny bursts of steam into the specific parts of your prostate that are causing the issues. This steam causes those cells to die off, and over time, your body naturally clears away this tissue. The result? Your prostate becomes smaller in the treated area, and this should help alleviate your symptoms.  Where and how is it done?: The procedure is typically done right here in the clinic. Depending on what's best for you, we might use local or general anesthesia to ensure you're comfortable during the treatment.  What about recovery?: One of the benefits of Rezūm is the quicker recovery time. Many men feel back to normal and can return to their daily activities within a few days. You might experience some temporary urinary symptoms, but these should improve as you heal.  Why consider Rezūm?: This treatment can reduce or even eliminate the need for medications for your enlarged prostate. Plus, most men find that it doesn't affect their sexual function, which can be a concern with some other treatments. Most importantly, it can provide relief from the uncomfortable symptoms of BPH.  We always want to ensure the best for your health, so it's crucial to discuss any concerns or questions you might have. We'll consider all options and choose the one that's best for your unique situation. | *EAU patient portal*:  Convective water vapour energy (WAVE) ablation, or the Rezum system  The surgeon passes a tool into the urethra and forces sterile water vapour or steam through it, into targeted parts of the prostate tissue. When the steam turns back into water, it causes the treated prostate cells to die. Over time, the body’s natural healing response removes the dead cells, thereby shrinking the prostate and enabling the urethra to open.  *AUA patient portal*:  uses water vapor (steam) to destroy prostate cells squeezing the urethra. This treatment can be done in a doctor’s office with local anesthesia or after you have taken a pill for pain. It uses a special handheld device with a needle at the end. It combines radiofrequency energy and water to create steam. The needle and steam cause rapid cell death. The body’s natural healing response then breaks down and removes the dead tissue, causing the prostate to shrink. |
|  | 74. What are the complications of REZUM? | REZUM is a treatment that uses steam to reduce the size of the prostate, which can help with urinary symptoms you might be experiencing. It's a less invasive option than some other treatments, but like any medical procedure, there are some potential side effects and risks to be aware of:  Blood in the urine: After the procedure, you might notice your urine is a little pink or has some blood in it. This is usually temporary and should clear up on its own.  Discomfort when urinating: Some men feel a burning sensation or discomfort when they urinate after the procedure, but this usually goes away after a short while.  Risk of infection: Just like after any procedure, there's a chance of getting an infection. If you notice symptoms like a fever or increased pain, let your doctor know.  Trouble urinating: In some cases, men find it hard to urinate after the treatment because of swelling. If this happens, you might need to use a catheter for a short time.  Needing to go to the bathroom often: You might find yourself needing to urinate more often right after the procedure. This usually gets better with time.  Changes in ejaculation: Some men notice that, after the procedure, when they ejaculate, the semen goes backward into the bladder instead of coming out. This isn't harmful but can be surprising.  Erection changes: A few men have reported changes in their erections after the procedure, but this is generally temporary.  Symptoms can come back: Over time, the prostate might grow again, and some of the symptoms could return.  Reactions to anesthesia: If you're given something to make you sleepy during the procedure, there's always a small risk of a reaction to that medication.  Tissue damage: There's a tiny chance that the procedure could accidentally harm the nearby tissues, but this is rare.  It might not solve all the symptoms: Some men don't feel a big difference in their symptoms after the procedure. It's different for everyone.  It's essential to weigh the potential benefits of getting relief from your symptoms with these possible side effects. Your doctor is here to guide you and answer any questions you might have. The goal is to choose the best option for you and your well-being. | *EAU patient portal*:  No information available  *AUA patient portal*:  You may have blood in your urine and need to use a catheter for a few days. Painful or frequent urination should go away within about two to three weeks. Sexual side effects, such as erectile dysfunction, are unlikely. |
|  | 75. What is the efficacy of REZUM? | Rezūm is a relatively new procedure that uses steam to treat the enlarged parts of your prostate. By doing this, it can help alleviate symptoms you might be experiencing, like frequent trips to the bathroom, difficulty starting your urine stream, or feeling like you can't completely empty your bladder.  How does it work?  Think of your enlarged prostate like a blockage in a water pipe. The prostate has grown larger and is pressing against the tube (urethra) where urine flows. Rezūm introduces tiny amounts of steam into the enlarged prostate. This steam damages the extra tissue causing the blockage. Over time, your body naturally clears away this damaged tissue, which can improve urine flow and reduce BPH symptoms.  Benefits of Rezūm:  It's a quick procedure, often taking less than 30 minutes.  There's minimal downtime. You can usually return to your regular activities within a few days.  One big plus for many men is that it has a lower risk of causing sexual problems compared to some other treatments.  It provides an option if you don't want to take daily medications or if those medications aren't working well for you.  Side Effects:  Like all medical procedures, there can be side effects. Some men might see a bit of blood in their urine, feel a burning sensation when they pee, or feel the need to go more often right after the procedure. These side effects typically don't last long and get better on their own.  Is it right for you?  Rezūm is one of several treatment options for BPH. There are other procedures and medications available. It's essential to discuss with your doctor about what's best for you based on your symptoms, the size and shape of your prostate, and other health considerations.  I hope this helps you understand the Rezūm procedure better. Please let me know if you have any questions or concerns. We're here to support you in making the best decision for your health. | *EAU patient portal*:  No information available  *AUA patient portal*:  Studies currently suggest that symptom improvement lasts for at least five years. |
|  | 76. What is the recurrence of benign prostatic enlargement after REZUM? | Rezūm is a treatment for BPH, which is an enlargement of the prostate that can cause uncomfortable urinary symptoms. We have good news from recent studies about this treatment:  Long-term Relief: Research shows that Rezūm can provide relief from urinary problems caused by BPH for up to five years. This means after getting this treatment, most men don't need any further surgery or medication for a long time.  Low Rate of Problems: In a study done in Italy, very few men (only about 1.5%) needed any additional treatment within 11 months after having Rezūm. This suggests that the treatment is quite effective.  Effective for Recurrent Symptoms: For men who had urinary symptoms come back after previous treatments, Rezūm proved to be a good option. After this treatment, most men didn't need to go back on medication for BPH. | *EAU patient portal*:  BPE cannot be completely cured and all medical and surgical treatments that are available focus on reducing the symptoms as far as possible. BPE symptoms and how bothersome they are can change over time. You may or may not decide to have further or more invasive treatment depending on your circumstances or how much the symptoms affect your daily life.  Your doctor will help you to make informed decisions about how you decide to treat your BPE and advise you of any new treatment options available so that your BPE is as well-managed as it can be, and you are able to enjoy your relationships and participate in all aspects of your life.  *AUA patient portal*:  No information available |
|  | 77. What is iTind? | It's a small device designed to help improve urinary symptoms caused by an enlarged prostate.  How does it work? iTind is temporarily placed inside the prostate. Over about a week, it gently expands, making tiny openings to help urine flow better.  Does it require a big surgery? No, it's minimally invasive. That means it's a simpler procedure compared to traditional surgeries. It's inserted using a thin tube and can often be done under local anesthesia, so you're awake but comfortable.  How long does it stay in? It stays inside the prostate for about 5-7 days. After that period, your doctor will remove it.  Is it effective? Many men have found relief from their urinary symptoms with the iTind. It can provide benefits for years in some cases.  Any side effects? Every procedure has potential risks, but many patients like iTind because it's less invasive, and there's no permanent implant left in the body. The risk of sexual side effects is also reduced compared to other treatments.  Is it right for me? It might be, but that depends on several factors like the size of your prostate and how severe your symptoms are. It's essential to talk with your doctor to decide the best option for you.  I hope that helps! If you have any more questions, don't hesitate to ask. | *EAU patient portal*:  iTind is a folded medical device which is inserted into the urethra, where it makes contact with the prostate. iTind stays in place for up to a week, during which time it unfolds, expands, and re-widens the urethra. The device is then removed, but the urethra stays re-shaped even after iTind has been taken out. This means that normal urine flow is restored and remains.  iTind is usually inserted under sedation and local anaesthetic.  *AUA patient portal*:  Temporary Implanted Prostatic Devices (TIPD) are placed in the prostatic urethra through a cystoscope and remain there for a period of about a week after which it is removed by the urologist. While in position, it functions to re-shape the urine channel. In re-shapeing the urethra, there is a modest lessening of the prostatic obstruction and thus a modest improvement in the symptoms of BPH and improved urine flow |
|  | 78. What are the complications of iTind? | Let me break down the possible side effects and complications for you in a straightforward way:  Blood in Urine: After the procedure, you might notice some blood in your urine. This is common and usually clears up on its own in a few days.  Infections: Anytime we do a procedure that involves the bladder or urine pathway, there's a small risk of getting an infection. If you notice symptoms like a burning sensation when you urinate or a fever, you should let us know.  Urgency to Urinate: Some men feel a strong, sudden urge to urinate or find they need to go more often for a short while after the procedure.  Discomfort: You may feel a bit of discomfort, especially when you urinate. This usually gets better on its own.  Possible Need for Further Treatment: Not everyone will have the same results from the iTind. Some might need another procedure or different treatments later on if their symptoms don't improve enough.  Device Movement: Very rarely, the device might move out of place before we intend to remove it. If this happens, we'd take care of it.  Strictures: Sometimes, a procedure can lead to narrowings called 'strictures' in the urine pathway, but this is generally less common with milder treatments like the iTind.  Sexual Side Effects: Some men experience changes in ejaculation after prostate procedures, like semen going backward into the bladder. This risk seems to be lower with iTind compared to other treatments.  Need for a Catheter: In very rare cases, you might need a temporary tube (catheter) to help you urinate if there's swelling blocking the flow.  Device-related Issues: Like with any device, there's a small chance of an unexpected issue or reaction. We'll be monitoring for these.  It's important to remember that every procedure has its pros and cons. The iTind is a less invasive option, which means it might have fewer side effects than more aggressive treatments. But it's essential to weigh the potential benefits against the risks. If you have any questions or concerns, please let me know. We're here to help and guide you through this decision. | *EAU patient portal*:  No information available  *AUA patient portal*:  It is a low-risk procedure that can be inserted in the OR or in the office. It may relieve bladder obstruction. TIPD poses a low risk of side effects, such as urinary tract infections, urinary incontinence and scarring in the urethra. Some men have symptoms that include frequent or intense urges to pass urine and a burning feeling when passing urine. |
|  | 79. What is the efficacy of iTind? | iTind is a small device doctors can use to help treat the problems men sometimes have when peeing because of an enlarged prostate. The prostate is a gland found in men that can get bigger as they age and press on the tube that carries urine out of the body, causing issues.  How does iTind work?  The doctor places the iTind device inside the tube in the prostate. Over a few days, this device gently expands, creating openings to make it easier for urine to flow out. After about a week, the doctor removes the device.  Is it effective?  Yes, studies have shown that many men feel better after having this treatment. Their symptoms, like having to go to the bathroom frequently or feeling like they can't empty their bladder completely, often improve.  Is it safe?  So far, the iTind seems to be safe. There are always risks with any medical procedure, but most men don't have significant problems with iTind. And a good thing about iTind is that it's less likely to cause sexual problems, which can sometimes happen with other prostate treatments.  Will the effect last long?  Based on what we know now, the benefits of the iTind treatment can last for several years. However, as with any new treatment, doctors are still gathering information to see how long the benefits last.  Remember, if you're thinking about getting the iTind treatment, it's essential to talk to your doctor. They can help you decide if it's the right choice for you and give you more details about the procedure. | *EAU patient portal*:  No information available  *AUA patient portal*:  There is a modest lessening of the prostatic obstruction and thus a modest improvement in the symptoms of BPH and improved urine flow. |
|  | 80. What is the recurrence of benign prostatic enlargement after iTind? | Certainly! When explaining the iTind procedure and its recurrence rate to a patient, it's important to use clear, simple language:  "iTind is a newer method for treating an enlarged prostate, a condition also known as Benign Prostatic Hyperplasia or BPH. In BPH, the prostate gland grows larger than normal, which can block or slow down the flow of urine.  The iTind treatment involves placing a small, temporary device inside the prostate. This device is made from a special material called nitinol. It's designed to gently expand and reshape the prostate tissue, which helps to improve urine flow. One of the benefits of iTind is that it doesn't involve cutting or removing any prostate tissue, which can lead to fewer side effects compared to traditional surgery.  As for how often the symptoms might come back after this treatment, it's a bit early to have precise numbers. iTind is relatively new, so doctors are still collecting long-term data. However, early results are promising. Many patients have seen good improvements with fewer side effects.  It's important to remember that every patient is different. The success of the iTind treatment and how long it lasts can depend on several factors, including how severe your symptoms are and your overall health. Your doctor can give you the best advice based on your specific situation. It's always a good idea to discuss all your options and any concerns you might have with them."  This approach ensures that the information is understandable and relevant to the patient's concerns and situation. | *EAU patient portal*:  BPE cannot be completely cured and all medical and surgical treatments that are available focus on reducing the symptoms as far as possible. BPE symptoms and how bothersome they are can change over time. You may or may not decide to have further or more invasive treatment depending on your circumstances or how much the symptoms affect your daily life.  Your doctor will help you to make informed decisions about how you decide to treat your BPE and advise you of any new treatment options available so that your BPE is as well-managed as it can be, and you are able to enjoy your relationships and participate in all aspects of your life.  *AUA patient portal*:  No information available |
|  | 81. What is prostate artery embolization? | Hi there! Let me explain prostate artery embolization, or PAE, to you.  You know how sometimes the prostate gland can grow larger as men age? This growth is not cancerous but can press on the tube that carries urine, causing problems like frequent trips to the bathroom or difficulty starting to urinate. This condition is called BPH, or benign prostatic hyperplasia.  What is PAE?  PAE is a way to treat these urinary symptoms without surgery. Instead of removing part of the prostate, we aim to reduce its size by decreasing its blood supply. Here's how:  The Procedure: We make a tiny puncture in the groin area and insert a small, flexible tube.  Using special X-ray equipment, we guide this tube to the blood vessels that supply the prostate.  We then release tiny particles through this tube, which block some of these blood vessels. By doing this, we reduce blood flow to the prostate, causing it to shrink a bit over time, which helps relieve your symptoms.  Why consider PAE?  It doesn't require a big surgical cut, just a tiny puncture.  You won't need to be fully asleep; just some local numbing.  Recovery time is generally quicker than with more extensive surgeries.  It tends to preserve sexual functions well.  Are there risks?  Like any medical procedure, there are some risks. Rarely, the particles might travel to an unwanted area, or there could be some discomfort or mild symptoms afterward. But we'll discuss all potential risks and benefits with you in detail before deciding anything.  In summary, PAE is a less invasive option to help alleviate the urinary problems caused by an enlarged prostate. If you think this might be right for you, or if you have more questions, let's chat further!  Remember, always consult with a medical professional to discuss any concerns or questions you might have about treatments. | *EAU patient portal*:  A vascular catheter is inserted into an artery in your groin. Using X-ray guidance, it is passed into the blood vessels that supply the prostate gland. Tiny plastic particles are injected into these vessels to reduce the prostate gland’s blood supply, which shrinks it. This procedure can be done under a local anaesthetic as an outpatient.  *AUA patient portal*:  Prostate Artery Embolization (PAE) is a radiologic procedure done by radiologists to block blood flow to the prostate to try to shrink the entire gland to improve symptoms. |
|  | 82. What are the complications of prostate artery embolization? | Prostate Artery Embolization, or PAE for short, is a procedure we use mainly to treat an enlarged prostate. Like any medical treatment, there are some potential side effects or risks, and I'd like to walk you through them so you're well-informed.  Where we insert the tube: Just like when you get a small bruise after an injection, there might be some swelling, bleeding, or even a tiny bump where we insert the catheter. Rarely, this site can get infected, but we take precautions to minimize this risk.  Going off-course: Imagine a roadmap with many routes. Sometimes, the materials we use to block the blood vessels might accidentally go into a neighboring route, affecting other areas unintentionally.  Effects on the bladder: There's a tiny chance that our procedure might reduce the blood supply to the bladder, which could cause some discomfort or injury.  Effects on the rectum: Similarly, there's a small risk that the rectum might get a reduced blood supply, which could lead to discomfort.  Blood in urine: Some patients might notice their urine is pinkish or reddish after the procedure, but this usually settles on its own.  Difficulty in urinating: On rare occasions, you might find it tough to pee after the procedure. If that happens, we have ways to help, like inserting a small tube.  Pain in the groin: You might feel some discomfort or pain where we inserted the catheter, but it typically goes away after a short while.  Feeling unwell post-procedure: Some people feel a bit under the weather after the treatment with mild pain, fever, or nausea. It's like how some folks feel after a flu shot – it usually gets better on its own or with some basic medicines.  Sexual function: There's a minimal chance of experiencing changes in sexual function, but it's essential to know it's a possibility.  Infections: Just like after any procedure, there's a slight chance of getting an infection, but we'll provide you with antibiotics if that happens.  Allergic reactions: Rarely, some patients might have a reaction to the materials we use during the procedure, similar to how some people are allergic to certain medicines.  X-rays: We use X-rays to see what we're doing during the procedure, and while the exposure is minimal, it's still a form of radiation.  Remember, these are potential risks, and not everyone experiences them. We're here to ensure that the procedure goes smoothly, and we'll be monitoring you closely during and after the procedure. If you have any concerns or questions, please let me know. We're here to help! | No information available in EAU and AUA patient portals |
|  | 83. What is the efficacy of prostate artery embolization? | PAE is a procedure to help with urinary symptoms that some men experience due to an enlarged prostate. Instead of surgery, small particles are injected to block blood flow to certain parts of the prostate, which can help reduce its size and relieve symptoms.  What can you expect from PAE?  Symptom Improvement: Many men have felt better after PAE. They've reported less trouble urinating and fewer trips to the bathroom, especially at night.  Prostate Size: The procedure can reduce the size of the prostate, which is one reason symptoms might improve.  Sexual Function: Some treatments for urinary symptoms can affect sexual function. With PAE, there's generally a lower chance of experiencing issues like difficulty getting an erection or changes in ejaculation.  Are there risks?  Like all medical procedures, there are some risks. The most common side effects include mild pain, a temporary fever, or seeing some blood in the urine. Rarely, there could be more serious issues, but these are uncommon.  How does it compare with other treatments?  You might have heard of a procedure called TURP. It's a surgical treatment that's been used for a long time for urinary symptoms. PAE is less invasive than TURP, and while some studies say TURP might give better relief, others say the two are pretty similar in results.  How long does it last?  The benefits of PAE can last for several years, but it varies from person to person. Some men might need another treatment down the road.  Is PAE right for you?  Not every man is a good fit for PAE. It's essential to talk with your doctor about your specific situation, the size and shape of your prostate, and other health factors to decide the best treatment for you.  I hope this helps you understand PAE a bit better. Always discuss with your doctor to get a clear picture tailored to your health and needs. | *EAU patient portal*:  No information available  *AUA patient portal*:  No information available in EAU and AUA patient portalsThis newer procedure does not have long term data to understand its durability. PAE probably improves urinary symptoms, at least for short periods measured for less than a year. There is a shortage of substantial evidence to recommend PAE over more widely available minimally invasive therapies for the routine treatment of LUTS, but there is evidence showing a short-term benefit of PAE compared to observation in a very select patient population. |
|  | 84. What is the recurrence of benign prostatic enlargement after prostate artery embolization | Prostate artery embolization (PAE) is a treatment for an enlarged prostate, a condition we also call benign prostatic hyperplasia (BPH). This treatment is less invasive compared to traditional surgery, which means it's generally easier on your body.  Now, about the chances of your enlarged prostate symptoms coming back after PAE:  Effectiveness of PAE: Many patients have found relief from their BPH symptoms after undergoing PAE. It's shown to be quite effective in reducing problems like difficulty in urinating or needing to go frequently.  Recurrence of Symptoms: Like with any medical treatment, there's a chance that symptoms might come back over time. The good news is, for many people, the relief from PAE lasts for several years. But, this can vary from person to person.  Factors Affecting Recurrence: Several things can influence whether your symptoms might return after PAE. This includes your age, how severe your BPH was before the treatment, and the expertise of the doctor who performed the procedure.  Staying Informed: Since PAE is a relatively new treatment, doctors are still learning about its long-term effects. It's important to stay in touch with your doctor and report any changes or return of symptoms.  Personalized Advice: Finally, remember that every person is different. Your doctor can give you the most accurate information based on your specific health situation.  In summary, PAE is a promising treatment for BPH with many patients experiencing long-lasting relief. However, there's a chance that symptoms could return, and this varies from person to person. It's important to keep an open line of communication with your healthcare provider to manage your health effectively. | No information available in EAU and AUA patient portals |
|  | 85. What is transurethral microwave thermotherapy? | Benign Prostatic Hyperplasia, or BPH, is a common condition where the prostate gland enlarges as men get older. This can cause trouble with urination, like needing to go more often or having a weak stream.  TUMT is a treatment we use for BPH. It's less invasive than surgery, which means it's simpler and generally safer. Here's how it works:  The Procedure: We use a special tube, called a catheter, which goes into your urethra (the tube you urinate through). Attached to this catheter is a tiny microwave device.  How It Works: This device sends out microwave energy. This energy heats up the enlarged part of your prostate. The heat destroys the extra tissue that's causing the problem, making it easier for you to urinate.  Safety Measures: While we do this, the catheter also circulates cool water. This keeps the heat from damaging the rest of your urinary tract.  What to Expect: You don't need to stay in the hospital for this. It's done on an outpatient basis, which means you can go home the same day. Most people find that the recovery is quicker and easier than with traditional surgery.  After the Procedure: Right after the procedure, you might feel some irritation when you urinate, or find it hard to urinate. This is temporary.  Benefits: The big advantage of TUMT is that it's less risky than surgery, especially when it comes to bleeding. And you can get back to your regular life quicker.  Considerations: While TUMT is effective, there's a chance you might need more treatment in the future. It's also possible, though rare, to have side effects like trouble with erections.  It's a good option for many men with BPH, especially if they're looking for a treatment that's less invasive than surgery. We can talk more about whether it's right for you, considering your health and how much your symptoms are bothering you. | No information available in EAU and AUA patient portals |
|  | 86. What are the complications of transurethral microwave thermotherapy? | Imagine your prostate gland is like a small doughnut, and the urethra (the tube that urine flows through) is like a straw running through its hole. As men get older, this "doughnut" can get bigger, making it hard for urine to pass through the "straw." TUMT is a procedure we use to help ease this problem.  Here's what happens in TUMT:  We use a special device that goes through the urethra to the prostate.  This device sends out microwave energy to heat and shrink the part of the prostate that's blocking urine flow.  Now, just like any medical procedure, there are possible side effects. Think of these like the bumps you might encounter on a road trip:  Urinary Issues: After the treatment, you might feel like you need to go to the bathroom more often, or it might feel a bit uncomfortable when you do. Sometimes, you might have trouble passing urine right after the procedure, but this usually gets better.  Irritation in the Pee Tube: The heat from the treatment might irritate the inside of your urethra (the pee tube). You might feel a burning sensation or see a little blood, but this usually goes away on its own.  Infection Risk: Just like with any procedure, there's a small chance of getting an infection. If you feel feverish, or if peeing becomes really painful or looks cloudy or bloody, let us know right away.  Changes in Sexual Function: Some men might notice changes in how they function sexually. This doesn't happen to everyone, but it's something to be aware of.  Retrograde Ejaculation: This is a fancy term for when semen goes into the bladder instead of coming out the usual way during ejaculation. It sounds odd, but it's generally not harmful and doesn't affect the feeling of sexual pleasure.  Narrowing of the Urethra: Sometimes, scar tissue can form and make the urethra narrower, which can make peeing difficult again.  Might Need More Treatment: In some cases, TUMT doesn’t completely fix the problem, and we might need to consider other treatments.  Remember, these side effects don't happen to everyone. It's important to talk about your specific situation and any concerns you have. We're here to make sure you get the care that's best for you! | No information available in EAU and AUA patient portals |
|  | 87. What is the efficacy of transurethral microwave thermotherapy? | Transurethral Microwave Thermotherapy (TUMT) is a treatment that uses microwave energy to reduce urinary symptoms caused by an enlarged prostate, a condition also known as benign prostatic hyperplasia (BPH). It's done as an outpatient procedure, meaning you don't need to stay in the hospital overnight.  Safety and Side Effects:  This procedure is generally safe and has a low risk of causing side effects. It's less invasive compared to some other prostate treatments.  Effectiveness:  TUMT is quite good at improving symptoms like difficulty in urination. However, it might not be as effective as some surgical methods, like the procedure called TURP (Transurethral Resection of the Prostate).  Benefits Regarding Sexual Function:  A significant advantage of TUMT is that it's less likely to affect your erectile function, which can be a concern with some other prostate treatments.  Retreatment Possibility:  One thing to keep in mind is that there's a chance you might need to undergo the treatment again in the future. TUMT has a higher rate of retreatment compared to some surgical options.  Comparing with Other Treatments:  In comparison to TURP, which is a more common surgical procedure for BPH, TUMT is slightly less effective in reducing symptoms and might require additional treatments later on. However, it's safer in terms of serious side effects that can occur during or after surgery.  In summary, TUMT is a safe, less invasive option that can effectively improve urinary symptoms due to an enlarged prostate. It's particularly good if you're concerned about maintaining sexual function. However, keep in mind that you might need more treatments in the future compared to some surgical options. It's always best to discuss all your options and concerns with your doctor to make an informed decision. | No information available in EAU and AUA patient portals |
|  | 88. What is the recurrence of benign prostatic enlargement after transurethral microwave thermotherapy? | The recurrence of benign prostatic hyperplasia (BPH) symptoms after transurethral microwave thermotherapy (TUMT) varies, depending on various factors such as the initial severity of BPH, the patient's overall health, and the effectiveness of the TUMT procedure itself.  Transurethral Microwave Thermotherapy (TUMT) is a minimally invasive procedure used to treat the symptoms of BPH. It uses microwave energy to heat and destroy excess prostate tissue, thereby relieving urinary symptoms. The procedure is generally well-tolerated and can be an alternative to more invasive surgeries.  Regarding recurrence rates:  Effectiveness and Duration: TUMT has been found to be effective in reducing BPH symptoms. However, the relief provided by TUMT may not be as long-lasting as more invasive procedures like transurethral resection of the prostate (TURP). Some patients may experience a recurrence of symptoms several years after TUMT.  Factors Influencing Recurrence: The likelihood of BPH symptoms recurring after TUMT can be influenced by factors such as the size of the prostate, the extent of the microwave treatment, and the patient's age and overall health.  Long-Term Outcomes: Studies have shown varied long-term outcomes. In some cases, additional treatments, either repeat TUMT or alternative therapies, may be required if symptoms recur or persist.  Follow-Up Care: Regular follow-up is important to monitor for symptom recurrence and to manage any ongoing urinary issues.  It's important for patients to discuss with their healthcare providers the potential risks and benefits of TUMT, including the likelihood of symptom recurrence, to make an informed decision about their treatment options. | No information available in EAU and AUA patient portals |
